# Numerical investigation of ultrasound-driven bubble-induced deformation of spherical solid cells

**DOI:** 10.1016/j.ultsonch.2026.107885

**Published:** 2026-05-12

**Authors:** Jaesung Park, Gihun Son

**Affiliations:** Research Institute for Smart Design & Manufacturing Technology, Department of Mechanical Engineering, Sogang University, 35 Baekbeom-ro, Mapo-gu, Seoul 04107, South Korea

**Keywords:** Ultrasound, Bubble collapse, Liquid jet, Cell deformation, Perforation

## Abstract

Ultrasound-driven bubble motion and the associated viscoelastic spherical cell deformation are studied within a coupled level set and volume-of-fluid (CLSVOF)-based compressible three-phase framework, in which solid deformation is handled through a full Eulerian description. The numerical simulations demonstrate various bubble–cell interaction phenomena, including inverted cone- and mushroom-shaped bubbles, droplet-shaped cells, liquid-jet formation, and bidirectional axial bubble splitting, by systematically varying the elastic shear modulus and initial bubble–cell distance. Effective bubble–cell interaction occurs when the initial bubble–cell distance falls below the maximum expansion radius of a free bubble under identical ultrasonic conditions. The potential cell damage mechanisms induced by ultrasound-driven bubble motion are shown to depend strongly on the shear modulus. The cell deformation is further analyzed as a function of cell size and ultrasonic pulse amplitude, revealing that cell size significantly influences cell deformation through its effect on the effective repulsion and attraction surface areas. The present results highlight that large cell deformation associated with potential cell disruption or sonoporation can be effectively regulated, for cells with a given size and shear modulus, by adjusting the initial bubble–cell distance, while for relatively stiff cells (G≥1MPa) such regulation shifts the critical cell size at which liquid-jet-induced deformation may lead to cell damage. It should be noted that the present analysis is limited to the first oscillation cycle (expansion–collapse–jet impact), as post-collapse dynamics do not achieve full grid convergence. Consequently, multi-cycle bubble dynamics and cumulative cell deformation are not considered in this study.

## Highlights

• Ultrasonic bubble growth and collapse drive spherical cell deformation.
• Shear modulus determines bubble collapse dynamics and deformation regimes.
• Cell size affects bubble–cell interaction through repulsion and attraction areas.
• Critical bubble–cell distance is identified for large compressive cell deformation.
• Ultrasonic pulse amplitude enhances liquid-jet velocity and cell damage potential.

## 1 Introduction

Under ultrasonic excitation, pre-existing bubbles undergo expansion and collapse, accompanied by shock-wave generation and high-velocity liquid jetting. These collapse-induced phenomena can exert mechanical loads on the surrounding medium, leading to significant forces and deformation in nearby solid cells. Owing to such effects, ultrasound-driven bubble dynamics have been widely utilized in various engineering applications, including wastewater treatment [1], ultrasonic cleaning [2], and targeted therapeutic techniques [3], [4]. In wastewater treatment, the shear stresses generated by collapsing cavitation bubbles must be sufficiently strong to effectively disrupt bacterial or algal cells [1]. In ultrasonic cleaning processes, liquid jets produced by bubble collapse must be capable of removing solid particles from semiconductor wafers while maintaining minimal pattern damage on the substrate [2]. In targeted drug delivery and cancer immunotherapy, the mechanical forces induced by bubble oscillation and collapse must be carefully regulated to minimize unintended biological damage, such as hemolysis [5], while remaining sufficiently strong to induce effective deformation or damage of tumor cells [3]. A fundamental understanding of cavitation bubble dynamics and their interaction with deformable solid cells is essential for optimizing a wide range of ultrasound-based applications.

Several experimental studies [6], [7], [8] have examined how cavitation bubbles affect deformable solid cells, including biological cells and bacteria. Nejad et al. [6] reported that acoustically excited lipid-stabilized bubbles induced pronounced deformation of histiocytic lymphoma cells with an initial radius of $R_{co}=9\;\mu m$ and transient membrane poration when the bubbles approached the cells. Zhou et al. [7] reported observations of a laser–generated bubble collapsing near a Xenopus oocyte cell ($R_{co}=400\;\mu m$) and showed that membrane poration became effective at an initial bubble–cell gap of approximately 1.5 times the free-bubble maximum radius. Li et al. [8] investigated microjet-induced membrane poration of myeloma cells with $R_{co}=10\;\mu m$ trapped in a microfluidic chip during the collapse of laser-generated bubbles, and demonstrated that the depth of drug uptake increased as the initial bubble–cell distance decreased. These experimental studies consistently demonstrate that the initial bubble–cell distance plays a critical role in cell deformation and membrane perforation, emphasizing the need for accurate control of bubble formation and collapse processes. Nevertheless, in ultrasonic cavitation environments, precise control of bubble nucleation and size remains inherently challenging [9], [10], and experimental investigations of bubble–cell interactions under such conditions are still relatively scarce.

Many numerical investigations [11], [12], [13], [14] on bubble–solid cell interactions under ultrasonic fields have been performed by treating the surrounding cell as a spherical droplet and representing its elasticity through surface tension effects. Gracewski et al. [11] analyzed ultrasound-driven bubble behavior in close proximity to deformable solids by applying the boundary element method (BEM). Guo et al. [12] further examined ultrasound-driven microbubble collapse near red blood cells using BEM and reported enhanced cell deformation with decreasing ultrasound frequency or initial bubble size. The above studies considered viscous and surface tension effects to play a minor role; however, these effects may become important for small bubble sizes under ultrasonic conditions [15]. Pan et al. [13] investigated cell penetration induced by liquid jets by employing the level-set method while varying cell viscosity and acoustic peak pressure, and reported that jet acceleration and penetration depth increased with the number of collapsing bubbles. More recently, Hong and Son [14] numerically analyzed the interaction between two microbubbles and a cell under ultrasonic conditions using the level-set method, demonstrating that the size ratio and initial inter-bubble distance are key parameters governing liquid jet acceleration. Despite these studies, droplet cell models that regard elastic features as surface tension–like effects have fundamental limitations in capturing the viscoelastic behavior of cells [16] and in accurately predicting the later stages of cell perforation [13].

Some numerical studies [17], [18], [19], [20] have computed elastic stress in cells near collapsing bubbles by adopting Lagrangian approaches for more realistic descriptions of solid deformation. Zevnik and Dular [17] modeled a spherical shell structure using Lagrangian nodal-based equations of motion and coupled them with the surrounding fluid solution to compute ribosome cell deformation in the vicinity of a collapsing bubble. Firly et al. [19] employed an arbitrary Lagrangian–Eulerian-based reference frame to describe the solid domain and solved the corresponding governing equations using the finite element method (FEM), through which they identified damage mechanisms in various metals and polymer substrates induced by liquid jets. Moon et al. [20] computed the underlying viscoelastic solid response using FEM and analyzed the effects of liquid jets on surrounding tissue deformation by varying the solid shear viscosity. Despite these studies employing Lagrangian-based approaches that are widely used for describing solid deformation, such methods generally suffer from mesh distortion and the need for mesh regeneration when large deformations occur, which leads to a significant increase in computational cost [21], [22].

In recent years, full Eulerian approaches [23], [24], [25], [26] have been increasingly adopted to investigate interactions between biological cells and cavitation bubbles, owing to their advantages in handling large solid deformations without mesh regeneration [21], [22]. Koukas et al. [24] computed incident shock-induced cavitation bubble collapse and the resulting penetration of surrounding elastic tissue by resolving both the solid and fluid responses within a fixed Eulerian grid. Shams et al. [25] extended their previous numerical framework [24] by incorporating hyperelastic constitutive behavior to effectively capture large soft tissue deformation and the associated tensile-driven tissue injuries. These computational frameworks have been successfully applied to various lithotripsy-related situations, where viscous and surface tension effects are generally considered to be minor. However, such frameworks remain limited in capturing the delayed deformation of viscoelastic materials in response to stress loading and release [20], and under acoustic cavitation conditions surface tension and viscous effects must be considered crucial due to the size of pre-existing nuclei or microbubbles [15]. More recently, Park and Son [26] numerically investigated ultrasonic cavitation bubble dynamics and viscoelastic tissue deformation by developing a level-set-based full Eulerian approach. They examined tissue deformation mechanisms by considering variations in acoustic impedance, elastic modulus, and bubble–tissue distance, and determined the parameter regimes associated with pronounced tissue deformation. Nevertheless, to the best of the authors’ knowledge, simulation studies on ultrasound-induced bubble motion near a spherical viscoelastic solid cell have not yet been reported.

In this work, we extend our previous coupled level set and volume-of-fluid (CLSVOF)-based framework to simulate ultrasound-induced cavitation bubble behavior near a spherical viscoelastic cell using a fully Eulerian approach proposed by Sugiyama et al. [23]. The liquid, gas, and solid phases are all treated as compressible media and described using the van der Waals (VDW) and Tait equations. Bubble collapse dynamics near a spherical cell with a large elastic modulus, which behaves nearly as a rigid solid, are first analyzed as a function of the initial bubble–cell distance. Subsequently, ultrasonically excited bubble behavior and liquid jet formation mechanisms are systematically compared among rigid, deformable, and soft cells by varying the elastic shear modulus. Furthermore, the influences of cell size on liquid jet generation and the associated cell perforation mechanisms are investigated for both rigid and deformable cells. Based on these analyses, the effective bubble–cell distance required to induce large cell deformation is numerically examined as a function of bubble size and elastic properties.

## 2 Numerical analysis

**Fig. 1:**
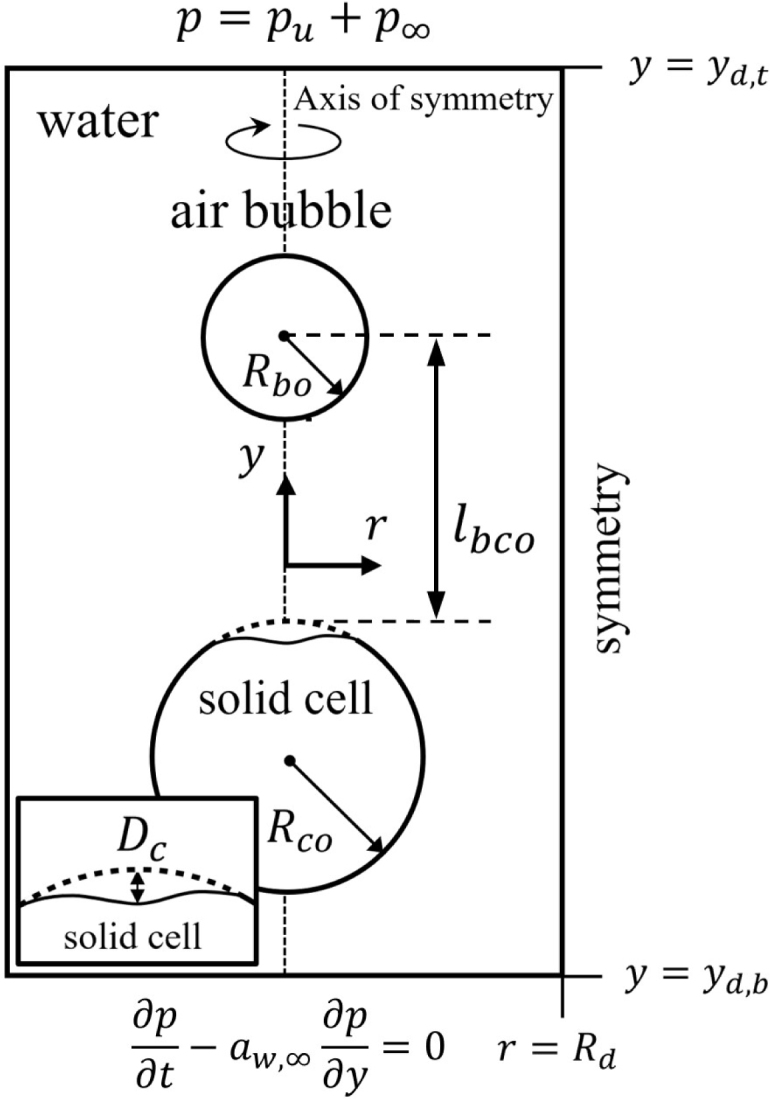
Schematic illustration of ultrasound-driven bubble motion and spherical solid cell deformation.

Our previously developed CLSVOF method [27] is extended to account for ultrasonic cavitation effects on a spherical viscoelastic cell, enabling the simulation of bubble growth, collapse, and the resulting cell deformation, as illustrated in Fig. 1. In the present study, a single microbubble interacting with a single cell is considered in order to isolate the fundamental bubble–cell interaction mechanisms, as commonly adopted in previous numerical studies [16], [17], [24], [25], [28]. In practical ultrasound environments, multiple bubbles and cells may coexist, and bubble–bubble interactions such as clustering, coalescence, or mutual jetting have been reported to significantly influence liquid jet formation and the resulting cell or tissue deformation [13], [14], [29].

The spherical cell is treated as a viscoelastic solid to reflect realistic biological situations, in contrast to earlier numerical studies on bubble–cell interactions where a compressible liquid representation was adopted for the cell [11], [12], [28]. The simulations assume axisymmetric behavior for both the fluid and the solid phases. All phases are assumed to be compressible fluids or solids; accordingly, the material properties of the solid cell, air bubble and water are assumed to be constant except for density. To account for compressibility effects in the water and the solid cell, the pressure $p_{w/s}$ is evaluated using the Tait equation, given as follows:

(1) $$p_{s/w}=p_{s/w,\infty }+\Pi_{s/w}\left[{\left(\frac{\rho_{s/w}}{\rho_{s/w,\infty }}\right)}^{\gamma_{s/w}}-1\right]$$

Here, $\gamma_{s}=\gamma_{w}=7.15$ and $\Pi_{s}=\Pi_{w}=3.31\times 10^{8}\;\mathrm{MPa}$, with the water volume fraction within the cell taken to be approximately 80% [12], [14]. The pressure inside the compressible bubble, $p_{b}$, is evaluated from the VDW equation, enabling an effective description of the high pressures developed during bubble collapse [26], as given by

(2) $$p_{b}=\left(p_{\infty }+a^{*}\rho_{b,\infty }^{2}\right)\left(\frac{1-a^{**}\rho_{b,\infty }}{1-a^{**}\rho_{b}}\right)-a^{*}\rho_{b}^{2}$$

Here, $a^{*}=27R_{g}^{2}T_{c}^{2}/64p_{c}$, $a^{**}=R_{g}T_{c}/8p_{c}$, and $R_{g}=287\;\mathrm{J/kg}\;K$, where the critical state of the air bubble is indicated by the subscript c.

### 2.1 Unified governing equations including solid deformation

The governing equations describing the solid cell, bubble, and water phases are given below.

(3) $$\frac{\partial \rho_{f/s}}{\partial t}+\nabla \cdot \left(\rho_{f/s}u_{f/s}\right)=0$$

(4) $$\frac{\partial \rho u}{\partial t}+\nabla \cdot \left(u\rho u\right)=-\left(\nabla p+\sigma \kappa_{w}\nabla H_{b}\right)+\nabla \cdot \left[\left(1-\alpha_{s}\right)\tau_{f}+\alpha_{s}\tau_{s}\right]$$

where

(5) $$\tau_{f}=\left[\mu_{b}\alpha_{b}+\mu_{w}\left(1-\alpha_{b}\right)\right]\left(\nabla u+\nabla u^{T}-\frac{2}{3}\left(\nabla \cdot u\right)I\right)$$

(6) $$\tau_{s}=\mu_{s}\left(\nabla u+\nabla u^{T}-\frac{2}{3}\left(\nabla \cdot u\right)I\right)+\tau_{e}$$

(7) $$\kappa_{l}=\nabla \cdot \frac{\nabla \phi_{b}}{\left|\nabla \phi_{b}\right|}$$

(8) $$H_{b}=1\;\;\mathrm{if}\;\phi_{b}\geq 0$$

$$=0\;\;\mathrm{if}\;\phi_{b}<0$$

Here, $\tau_{f}$ represents the viscous stress of the fluid phases, $\tau_{s}$ denotes the viscoelastic stress of the solid phase, and $\tau_{e}$ represents the elastic stress. The parameter $\mu_{s}$ corresponds to the solid viscosity associated with viscous dissipation. By combining Eqs. (9)–(6), the momentum conservation equation can be rewritten in the following unified form:

(9) $$\frac{\partial \rho u}{\partial t}+\nabla \cdot u\rho u=-\left(\nabla p+\sigma \kappa_{w}\nabla H_{b}\right)+\nabla \cdot \left[\mu \left(\nabla u+\nabla u^{T}-\frac{2}{3}\left(\nabla \cdot u\right)I\right)+\alpha_{s}\tau_{e}\right]$$

where

(10) $$\rho =\rho_{s}\alpha_{s}+\left[\rho_{b}\alpha_{b}+\rho_{w}\left(1-\alpha_{b}\right)\right]\left(1-\alpha_{s}\right)$$

(11) $$\mu =\mu_{s}\alpha_{s}+\left[\mu_{b}\alpha_{b}+\mu_{w}\left(1-\alpha_{b}\right)\right]\left(1-\alpha_{s}\right)$$

(12) $$\tau_{e}=G\left(B-I\right)$$

Here, $\kappa_{w}$ denotes the curvature of the bubble interface, and $H_{b}$ represents the Heaviside function. $\phi_{f/s}$ and $\alpha_{f/s}$ denote the level-set function and the volume fraction of each phase f/s, respectively. In Eq. (12), the elastic stress $\tau_{e}$ is defined using a neo-Hookean constitutive relation involving the left Cauchy–Green deformation tensor B and the shear modulus G. In the present study, stress relaxation effects are not explicitly considered, corresponding to an effectively infinite relaxation time. This assumption is justified by the separation of time scales: biological soft materials typically exhibit relaxation times on the order of milliseconds to seconds, whereas the characteristic time scale of ultrasound-driven bubble growth and rapid collapse is on the order of sub-microseconds (<1μs). Under these conditions, the solid response can be reasonably approximated as hyperelastic. It should be noted that the elastic stress $\tau_{e}$ given in Eq. (12), is based on the neo-Hookean model, which is a special case of the Mooney–Rivlin model. This model is widely adopted to represent large deformation of biological materials and soft rubber-like solids, and has been successfully used in previous Eulerian simulations of cell and soft tissue deformation [22], [23], [30], [31]. Within the Eulerian framework, the evolution of B is described as follows [15], [23]:

(13) $$\frac{\partial B}{\partial t}+u\cdot \nabla B={\left(\nabla u\right)}^{T}\cdot B+B\cdot \left(\nabla u\right)$$

### 2.2 CLSVOF method for compressible multiphase flow

To track the solid, gas, and liquid interfaces while ensuring mass conservation, α and ϕ are advanced using an operator-splitting approach [27], [32] as follows.

(14) $$\frac{\partial \rho_{b/s}\alpha_{b/s}}{\partial t}+\nabla \cdot \rho_{b/s}\alpha_{b/s}u_{b/s}=0$$

(15) $$\frac{\partial \phi_{b/s}}{\partial t}+u_{b/s}\cdot \nabla \phi_{b/s}=0$$

Here, $\alpha_{b/s}$ given in Eq. (14) is computed based on the updated density $\rho_{b/s}$, which is obtained from the mass Eq. (3) using the ghost fluid method [33]. The volume flux in the convection term of Eq. (14) requires the swept volume by the interface over a time step Δt, which is determined from the interface normal $n=\nabla \phi_{b/s}/\left|\nabla \phi_{b/s}\right|$ and the distance $s_{b/s}=f\left(\phi_{b/s},\alpha_{b/s}\right)$ from a vertex to the interface [27]. The functional forms for the swept volume and the face distance $s_{f}$, which are explicitly determined from $\alpha_{b/s}$ and $\phi_{b/s}$, have been described in detail in our previous studies [27]. In general, the LS function $\phi_{b/s}$ advanced by Eq. (15) loses its signed-distance property; therefore, it is reinitialized based on the reconstructed interface as follows.

(16) $$\phi_{b/s}=s_{b/s}-\frac{1}{2}\left(\Delta r\left|n_{b/s,r}\right|+\Delta y\left|n_{b/s,y}\right|\right)\;\mathrm{if}\;0<\alpha_{b/s}<1$$

(17) $$\frac{\partial \phi_{b/s}}{\partial t^{*}}=\frac{\phi_{b/s}}{\sqrt{\phi_{b/s}^{2}+h^{2}}}\left(1-\left|\nabla \phi_{b/s}\right|\right)$$

Here, $t^{*}$ denotes an artificial time step, and Δr/y and $n_{b/s,r/y}$ represent the grid spacing and the interfacial normal vector components aligned with the r and y directions, respectively. Near the interface, $\phi_{b/s}$ is first directly reinitialized for $0<\alpha_{b/s}<1$ using the geometric relation given in Eq. (16), and the LS function $\phi_{b/s}$ is then reinitialized by iteratively solving Eq. (17).

**Fig. 2:**
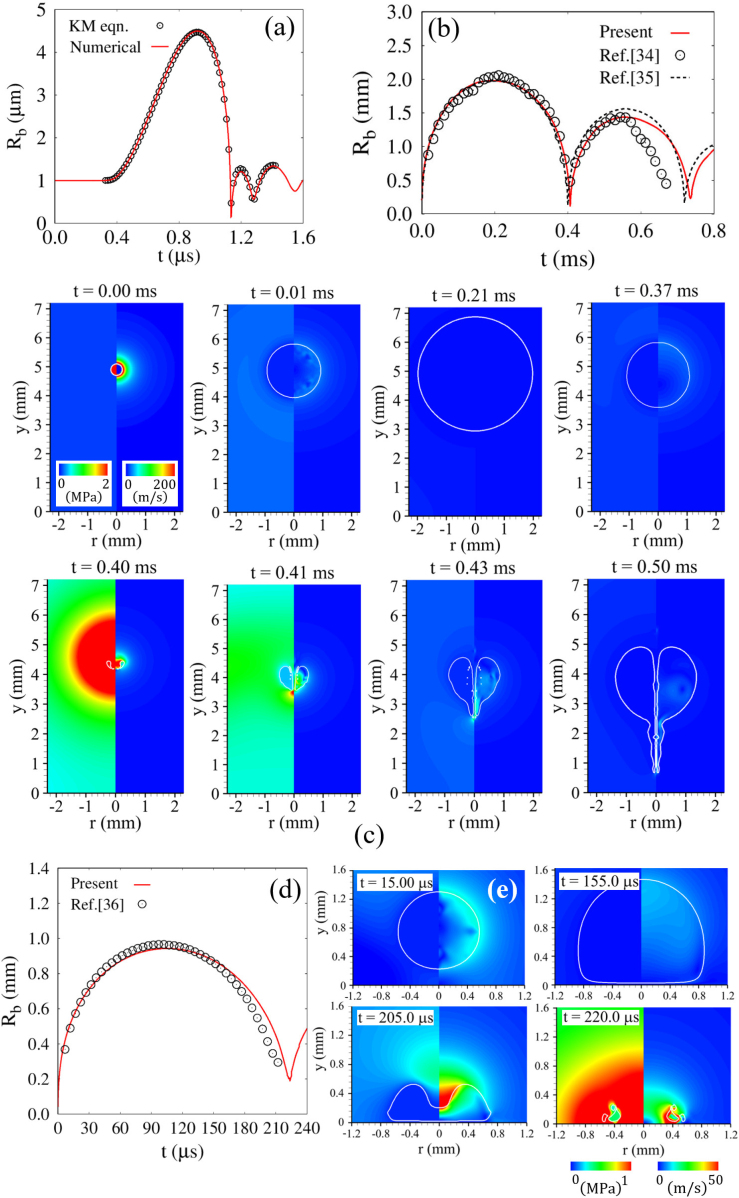
Comparison of the numerical predictions with (a) the Keller–Miksis equation; (b,c) previous experimental data [34] and numerical results [35]; (d,e) previous experimental data [36].

### 2.3 Model validation

To assess the predictive capability of the present numerical approach, validation studies were conducted for canonical single-bubble dynamics under well-established benchmark conditions. Initially, an air bubble with a radius of $R_{bo}=1\;\mu m$ is immersed in ambient water, and an ultrasonic pressure pulse, $p_{u}=-P_{u}\sin\left(2\pi f_{u}t\right)$, s imposed at the upper boundary ($y=Y_{d,t}$). Fig. 2(a) compares the present numerical results with the solution of the Keller–Miksis equation [14], which is widely used as a reference for spherically symmetric bubble dynamics. Here, the equivalent bubble radius is evaluated from the bubble volume $V_{b}$ as $R_{b}={\left(3V_{b}/4\pi \right)}^{1/3}$. As the negative pressure reaches the bubble interface, the bubble undergoes continuous expansion and attains its first maximum radius of $R_{b,max1}=4.49\;\mu m$ at t=0.918μs. It is noted that $R_{b,max}$ is used to normalize the initial bubble–cell distance $l_{bco}$. During the subsequent positive pressure phase, the bubble rapidly contracts and collapses at t=1.135μs, followed by rebound and subsequent oscillations. The temporal evolution of $R_{b}$ over the entire growth–collapse cycle shows good agreement with the Keller–Miksis solution, indicating that the present approach reasonably captures the combined effects of viscosity, surface tension, and liquid compressibility.

For near-wall bubble dynamics, validation is conducted using previously reported experimental and numerical studies [34], [35], [36]. Fig. 2(b) shows the temporal evolution of the equivalent bubble radius for the conditions reported by Wang [35]($R_{bo}=200\;\mu m$, ${\overset{˙}{R}}_{bo}=300\;\mathrm{m/s}$, $p_{bo}=5\;\mathrm{MPa}$), compared with experimental data from Ref. [34]. The present results show good agreement with both experimental and numerical data in terms of expansion and collapse dynamics. In this case, the initial conditions are $R_{bo}=0.2\;\mathrm{mm}$, ${\overset{˙}{R}}_{bo}=300\;\mathrm{m/s}$, $p_{bo}=5\;\mathrm{MPa}$, and the initial bubble–wall distance $l_{bco}=4.9\;\mathrm{mm}$, as described in Ref. [34]. Due to the initial pressure difference and interface velocity, the bubble undergoes rapid initial expansion and reaches $R_{b,max1}=1.98\;\mathrm{mm}$, which differs from the experimental value by approximately 1%, demonstrating quantitative agreement within a small margin of error. The subsequent collapse occurs over 0.212ms, which is qualitatively consistent with the previous numerical results [35]. During the 1st expansion–collapse cycle, the temporal variation of $R_{b}$ shows good agreement with both the experimental data [34] and previous numerical results [35]. During the 2nd rebound, the predicted bubble radius also follows the experimental trend, with the second maximum radius $R_{b,max2}=1.436\;\mathrm{mm}$, showing only a 0.48% deviation from the experimental value. The present results show good agreement with both experimental and numerical data in terms of expansion and collapse dynamics. In addition to the temporal evolution of the bubble radius, the numerical results for bubble expansion–collapse dynamics and liquid-jet formation are shown in Fig. 2(c). The bubble initially undergoes rapid expansion due to the high internal pressure, followed by contraction starting at t=0.212ms, and forms a liquid jet at t=0.40ms. The strong downward momentum induced by the jet drives the jet tip toward the rigid wall and causes an overall downward translation of the bubble. Overall, the predicted expansion–collapse behavior and jet formation process show good agreement with the visualization results reported in Ref. [34]. To further assess the applicability of the method in the microbubble regime, additional validation is performed for a smaller initial radius ($R_{bo}=41,\;\mu m$) and a higher internal pressure ($p_{bo}=1\;\mathrm{GPa}$), as shown in Fig. 2(d, e). The predicted radius evolution shows good agreement with the experimental trends reported in Ref. [36], and the simulation captures the overall oscillation behavior and collapse characteristics of the microbubble. Although some quantitative discrepancies are observed in the later stages of collapse, such differences are commonly reported in cavitation studies and are primarily attributed to uncertainties in experimental initial conditions and measurement limitations. Nevertheless, the present method successfully captures the essential features of bubble dynamics across both millimeter- and micro-scale regimes. These validation results support the capability of the present CLSVOF framework to reproduce key features of microbubble dynamics, including expansion, collapse, rebound, and near-wall interaction.

**Fig. 3:**
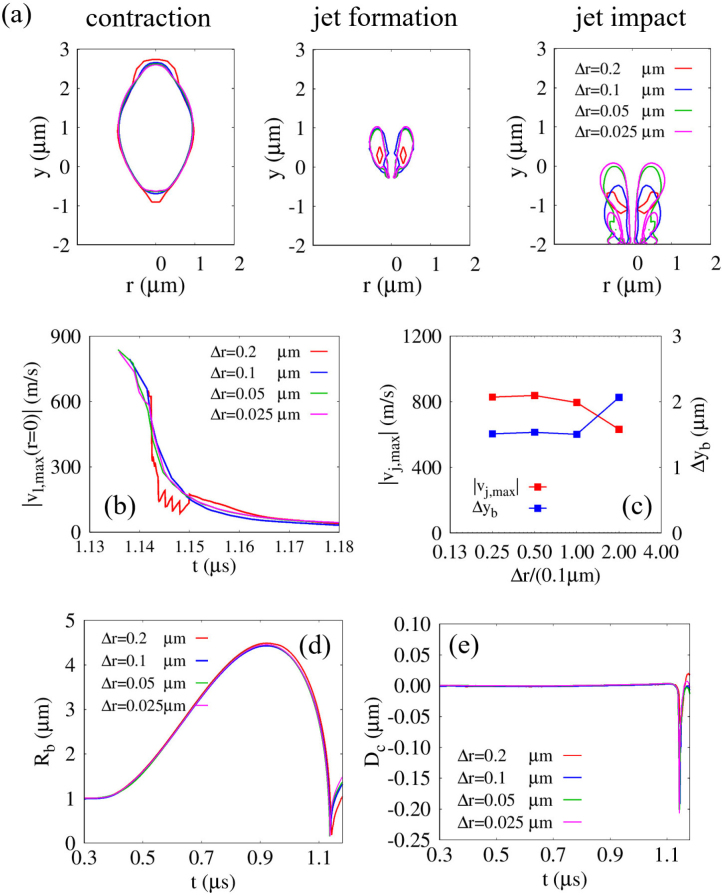
Grid convergence test for ultrasonic bubble motion and cell deformation under the same conditions as Fig. 5(b), with grid resolutions ranging from Δr=0.2μm to 0.025μm: (a) bubble interface at three characteristic stages—contraction (left), jet formation (center), and jet impact (right, shown for qualitative comparison due to increased sensitivity in the post-collapse regime); (b) time history of the maximum liquid velocity $v_{l,max}$ along the central axis; (c) maximum liquid-jet velocity; (d) averaged bubble radius $R_{b}$; and (e) cell deformation $D_{c}$. The time histories in (d) and (e) are presented up to t=1.18μs, corresponding to the pre-collapse and jet-impact stages where consistent grid convergence is observed.

To validate the liquid-jet formation induced by bubble collapse and the resulting cell deformation, preliminary computations are performed for ultrasonic bubble–cell interaction. As shown in Fig. 3, simulations are conducted under the same conditions as in Fig. 5(b) using four different grid resolutions, 0.025μm≤Δr≤0.2μm. Fig. 3(a) presents the bubble interfaces at three representative instants: the contraction phase (left), the onset of liquid-jet formation (center), and the jet impact stage (right). During the contraction and jet formation stages (left and center panels), the discrepancy between the bubble interfaces decreases progressively with grid refinement. In particular, the differences become negligible for Δr=0.1μm, especially between the finer grids (Δr=0.05μm and 0.025μm), convergence of the bubble dynamics and supporting the choice of Δr=0.1μm. In contrast, noticeable deviations in bubble shape are observed during the jet impact stage (right panel). This behavior is attributed to the increased sensitivity of the interface in the post-collapse regime, where small numerical perturbations can accumulate and be amplified. A similar trend is observed in the rebound dynamics shown in Fig. 3(d), where a slightly larger rebound radius appears for the finest grid (Δr=0.025μm). To clearly assess grid convergence, the time histories of $R_{b}$ and $D_{c}$ are presented up to t=1.18μs, which includes the bubble collapse and the subsequent jet-impact stage, as plotted in Fig. 3(d) and (e). In this time range, good agreement is observed across different grid resolutions, particularly for the first peak compressive deformation of $D_{c}$, which is the primary quantity of interest in this study. Beyond this point, the post-collapse dynamics become increasingly sensitive to small numerical perturbations due to the highly nonlinear interface evolution. As a result, noticeable discrepancies arise in the rebound regime, and this phase is therefore not considered for quantitative analysis. It is also noted that the convergence of $D_{c}$ is maintained over a longer duration than that of $R_{b}$. While $R_{b}$ is directly influenced by the instantaneous interface position, $D_{c}$ reflects the structural response of the cell, which integrates the fluid forcing over time and is therefore less sensitive to high-frequency variations. It is important to note that the contraction and jet formation stages are the key phases governing the jet strength, jet tip position, and the resulting cell deformation, which are the primary focus of this study. This is further supported by the convergence observed in Fig. 3(b) and (c), where the temporal evolution of the axial liquid velocity $\left|v_{l,max}\left(r=0\right)\right|$, the maximum jet velocity $\left|v_{j,max}\right|$, and the bubble displacement at collapse $\Delta y_{b}$ exhibit consistent behavior with decreasing Δr, particularly between Δr=0.05 and 0.025μm. These results indicate that, although complete geometric convergence of the interface is difficult to achieve in the highly nonlinear collapse regime, the primary quantities governing jet dynamics and cell deformation are sufficiently resolved for Δr≤0.1μm. The cell deformation $D_{c}$ also shows reasonable convergence within this range, particularly near the first peak compressive deformation, which is the main focus of this study. Complete grid convergence in the post-collapse regime is difficult to achieve due to the highly nonlinear and transient nature of the interface dynamics. Therefore, the present study focuses on the pre-collapse and jet-impact stages, where the key physical quantities are sufficiently resolved. Based on these results, Δr=0.1μm is selected as a compromise between computational efficiency and numerical accuracy.

## 3 Results and discussion

### 3.1 Bubble collapse near a rigid solid cell

In this work, a spherical solid cell and an air bubble immersed in ambient water at $T_{\infty }=20{}^{\circ}C$ and $p_{\infty }=1\mathrm{atm}$ are considered, as illustrated in Fig. 1. The material properties of air, water, and the solid cell used in the simulations are as follows: $\sigma =7.3\times 10^{-2}\;{\mathrm{kg/s}}^{2}$, $\Pi_{w}=\Pi_{s}=3.31\times 10^{8}\;\mathrm{Pa}$, $\mu_{w}=\mu_{s}=10^{-3}\;\mathrm{Pa}$, $\rho_{w,\infty }=\rho_{s,\infty }=998\;{\mathrm{kg/m}}^{3}$, $\mu_{b}=10^{-5}\;\text{Pa s}$, and $\rho_{b,\infty }=1.23\;{\mathrm{kg/m}}^{3}$. As the initial condition, a solid cell with an initial radius $R_{co}$ is located at (r,y)=(0,−7μm). A spherical microbubble with an initial radius $R_{bo}=1\;\mu m$ is placed at $\left(r,y\right)=\left(0,-7\;\mu m+l_{bco}\right)$, separated from the cell by the bubble–cell distance $l_{bco}$, within a cylindrical domain defined by $r<R_{d}=5897R_{bo}$ and $Y_{d,b}=-5897R_{bo}\leq y\leq Y_{d,t}=508R_{bo}$. A fine uniform mesh with Δr=Δy=0.1μm is employed in the region $r\leq 19.2R_{bo}$ and $-25.6R_{bo}\leq y\leq 19.2R_{bo}$ surrounding the bubble and the solid cell, while a coarser non-uniform grid is used in the remaining outer region. It is noted that the computational domain size defined by $R_{d}$ and $Y_{d,b}$ is sufficiently larger than four times the acoustic wavelength ($a_{w}/f_{u}$) to prevent reflections of pressure waves from disturbing the bubble motion [14], [37]. To minimize pulse reflection, a convective boundary condition, $\partial p/\partial t+a_{w,\infty }\partial p/\partial y=0$, is imposed at the lower boundary ($y=Y_{d,b}$) [14], [15]. It should be noted that multi-cycle ultrasound can intensify interference among collapse-induced shock waves, imposed acoustic waves, and reflected waves. As a result, simulations with multi-cycle ultrasound require significantly extended computational domains or additional wave-absorption treatments to prevent artificial interference from reflected waves. In the present study, a single-cycle pressure pulse is employed to isolate wave-interference effects and to enable a fundamental analysis of bubble–cell interaction.

To account for ultrasonic excitation, pressure boundary conditions are imposed at the upper boundary ($y=Y_{d,t}$) as given by

(18) $$p_{u}=-P_{u}\sin\left(2\pi f_{u}t\right)\;\;\mathrm{if}\;\;f_{u}t\leq N_{u}$$

Here, the parameters $P_{u}$, $f_{u}$ and $N_{u}$ denote the ultrasonic pressure amplitude, frequency, and number of cycles, respectively. In the simulations, $l_{bco}$, G, $R_{co}$, and $P_{u}$ are varied, whereas the number of cycles and frequency are fixed at $N_{u}=1$ and $f_{u}=1\;\mathrm{MHz}$, respectively. Note that $N_{u}=1$ corresponds to a single-cycle pulse, which is commonly adopted in previous numerical studies on bubble–cell interactions under ultrasonic conditions [11], [12], [14], [28]. In general, higher frequencies tend to lead to reduced bubble oscillation amplitude and weaker jetting, whereas lower frequencies may promote larger bubble expansion and more intense collapse dynamics [14], [15]. It should be noted that a systematic investigation of frequency effects on bubble–cell interactions would be an important direction for future research.

**Fig. 4:**
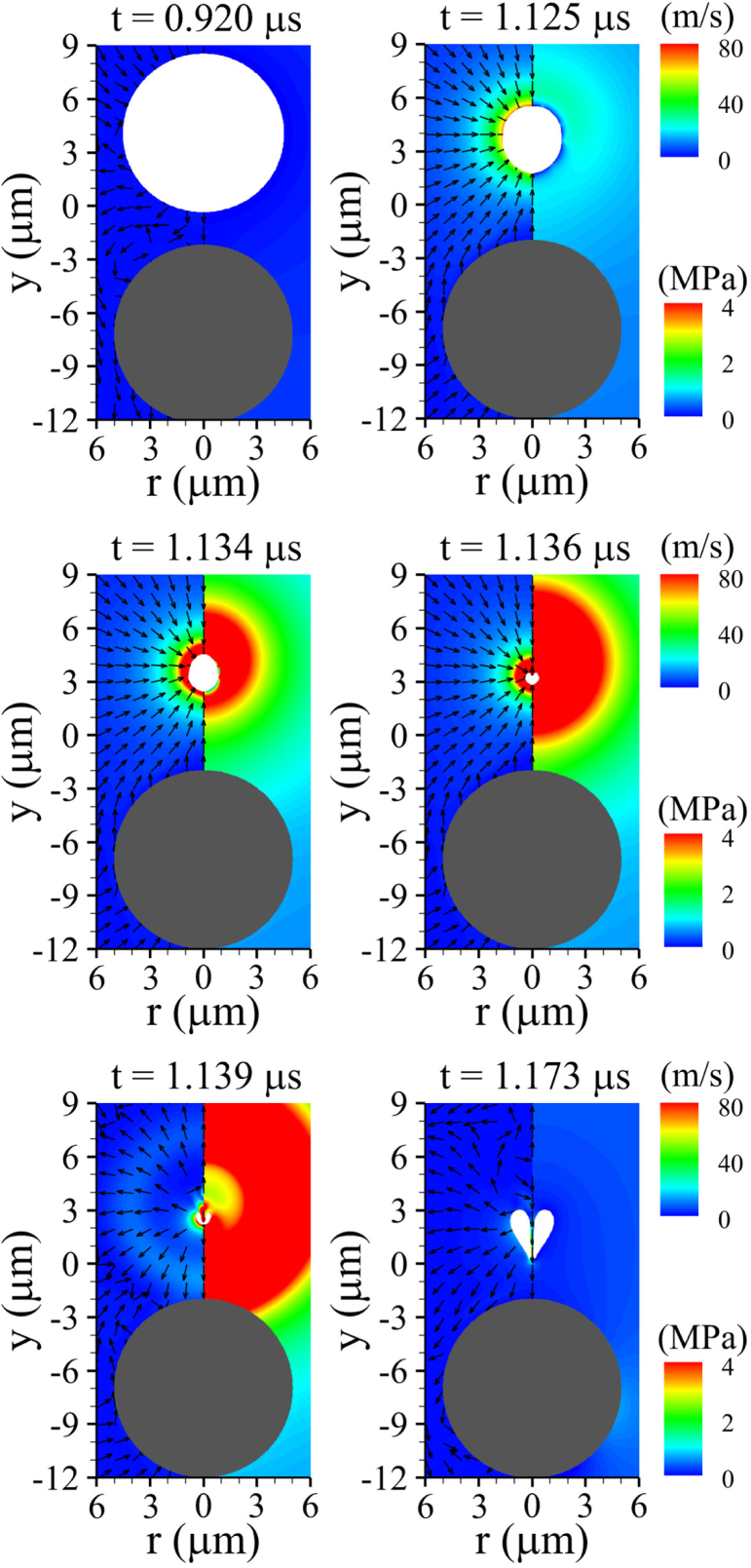
Liquid velocity (left) and pressure (right) fields during bubble collapse near a rigid spherical cell (G=100MPa) for $l_{bco}=6\;\mu m$, $R_{co}=5\;\mu m$, $P_{u}=0.3\;\mathrm{MPa}$, and $f_{u}=1\;\mathrm{MHz}$. The white and gray regions indicate the bubble and the solid cell, respectively.

Our numerical results are first presented for a rigid spherical cell with a large shear modulus. Fig. 4 illustrates the ultrasound-driven bubble contraction and collapse for G=100MPa, $l_{bco}=6\;\mu m>R_{b,max}^{o}$, $R_{co}=5\;\mu m$, and $P_{u}=0.3\;\mathrm{MPa}$. Here, $R_{b,max}^{o}=4.47\;\mu m$ denotes the maximum expansion radius of an isolated free bubble computed using the Keller–Miksis equation [14] under the same acoustic conditions. The acoustic pressure pulse generated at $y=Y_{d,t}$ travels through liquid water with speed $a_{w,\infty }$ and arrives at the bubble at $t=f_{u}Y_{d,t}=0.303\;\mu s$, thereby triggering bubble expansion. Throughout the subsequent expansion stage, the bubble induces an outward source flow from its interface, displacing the surrounding liquid and pushing the nearby rigid cell away. The bubble attains its maximum expansion as seen at t=0.920μs, after which it undergoes contraction driven by the compressive surrounding pressure field during the positive pressure pulse period (0.803μs<t<1.303μs). As observed at t=1.125μs, this contraction generates a sink-type flow directed toward the bubble center, producing a hydrodynamic attractive force that induces upward rigid-body motion of the nearby cell without noticeable deformation. As the bubble further contracts, the presence of the rigid cell beneath the bubble causes slight asymmetry between the upper and lower flow fields; however, because the bubble and the cell are separated by a relatively large gap, the interaction is limited, as shown for 1.134μs≤t≤1.136μs. Upon reaching its minimum volume, the bubble releases a strong internal pressure into the surrounding liquid in the form of a shock wave, and subsequently re-expands into a highly distorted shape at t=1.139μs. The distorted bubble then undergoes centerline penetration by the upstream liquid flow during its re-expansion, whose momentum leads to the downward bubble motion observed at t=1.173μs. Although this momentum applies a downward force to the rigid cell surface, the resulting downward motion of the rigid cell remains negligible due to the still large surface-to-surface separation and the insufficient momentum associated with the weak liquid jet. Note that the distance $l_{bco}$ is noticeably larger than $R_{b,max}^{o}$, implying that the bubble–cell interaction may become more prominent if the bubble is controlled to be positioned more closely to the cell.

**Fig. 5:**
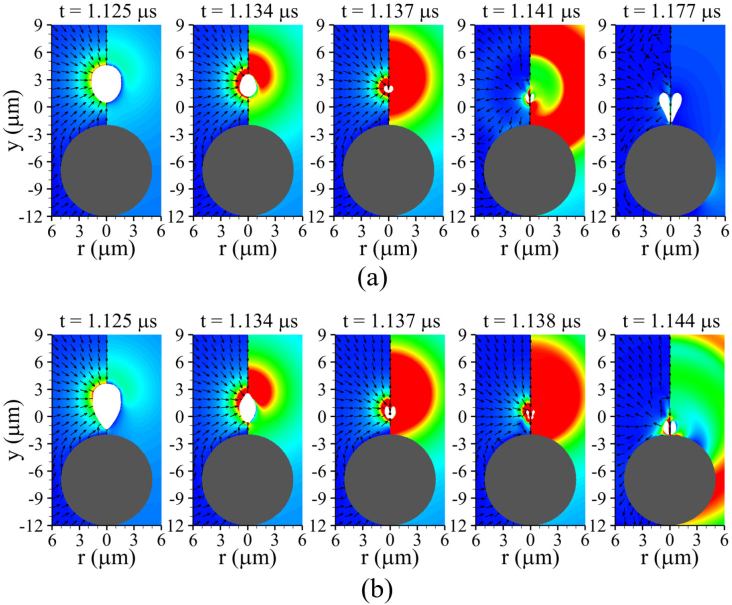
Liquid velocity (left) and pressure (right) fields during bubble collapse near a rigid spherical cell (G=100MPa) for (a) $l_{bco}=5\;\mu m$ ($l_{bco}>R_{b,max}^{o}$), showing weak interaction due to insufficient jet formation, and (b) $l_{bco}=4\;\mu m$ ($l_{bco}<R_{b,max}^{o}$), showing strong bubble–cell interaction with pronounced jet impact, while all other parameters are kept the same as in Fig. 4. The white and gray regions indicate the bubble and the solid cell, respectively.

### 3.2 Effect of initial bubble-cell distance

Fig. 5(a) shows the results obtained for a configuration where the bubble is initially positioned at $l_{bco}=5\;\mu m$ from the rigid cell surface, with all other parameters unchanged. During the early stage of collapse, bubble dynamics together with the surrounding liquid velocity and pressure fields at t=1.125μs remain similar to those observed for $l_{bco}=6\;\mu m$ in Fig. 4. With ongoing collapse, the flow behavior shows a gradual deviation relative to the $l_{bco}=6\;\mu m$ case, accompanied by an increasingly pronounced asymmetry in velocity and pressure distributions between the upper and lower portions of the bubble over 1.134μs≤t≤1.137μs. This asymmetric feature enhances the liquid flow penetrating through the collapsed bubble, leading to an increase in jet velocity. The resulting liquid momentum is subsequently redirected, causing the bubble to move downward toward the cell, as observed over 1.141μs≤t≤1.177μs. The bubble moves into close proximity to the cell at t=1.177μs; however, the jet momentum at this time remains insufficient to induce an appreciable downward rigid-body motion of the cell. It should be noted that for $l_{bco}>R_{b,max}^{o}$, no significant bubble–cell interaction is observed; however, as shown at t=1.177μs, the collapsing bubble translates toward the cell, reducing the bubble–cell distance relative to its initial position. This suggests that, under multi-cycle excitation, the bubble may re-expand from a reduced separation distance, leading to enhanced bubble–cell interaction during subsequent cycles. Although this is beyond the scope of the present study, it suggests that under multi-cycle acoustic conditions, enhanced liquid-jet formation and increased cell deformation may occur during the second collapse.

For a shorter bubble–cell distance of $l_{bco}=4\;\mu m$, the simulation results are shown in Fig. 5(b). This case corresponds to the condition with $l_{bco}<R_{b,max}^{o}$. Compared with the early collapse stage observed in Figs. 4 and 5(a), the bubble exhibits an inverted cone-like shape with a highly curved interface near the solid surface at t=1.125μs, resulting from the strong retardation of the contracting bubble’s lower surface. This behavior agrees with earlier experimental findings [38], in which laser-induced cavitation bubbles were observed to grow and collapse near a fixed spherical particle using high-speed imaging. During the subsequent contraction, the pronounced asymmetry between the flow fields above and below the bubble observed at t=1.134μs leads to a highly distorted bubble shape and a collapse location very close to the cell surface, as shown at t=1.137μs. The bubble migrating toward the cell surface is penetrated as it reaches its minimum volume, resulting in liquid jet emergence at t=1.138μs. In contrast to the $l_{bco}=6\;\mu m$ case, in which the bubble is pierced by the surrounding liquid inertia during the subsequent rebound, the resulting liquid jet is substantially stronger. The large jet velocity and its associated momentum allow the jet tip to reach the cell surface, as shown at t=1.144μs, and the resulting impact induces a downward rigid-body motion of the cell accompanied by a slight localized deformation. This trend is consistent with previous experimental and numerical observations [39] reporting an increased pushing velocity of a suspended sphere when a spark-induced cavitation bubble is generated closer than its maximum radius. These results indicate that when $l_{bco}$ is sufficiently small compared to $R_{b,max}^{o}$, both bubble–cell interaction and liquid jet formation become pronounced.

**Fig. 6:**
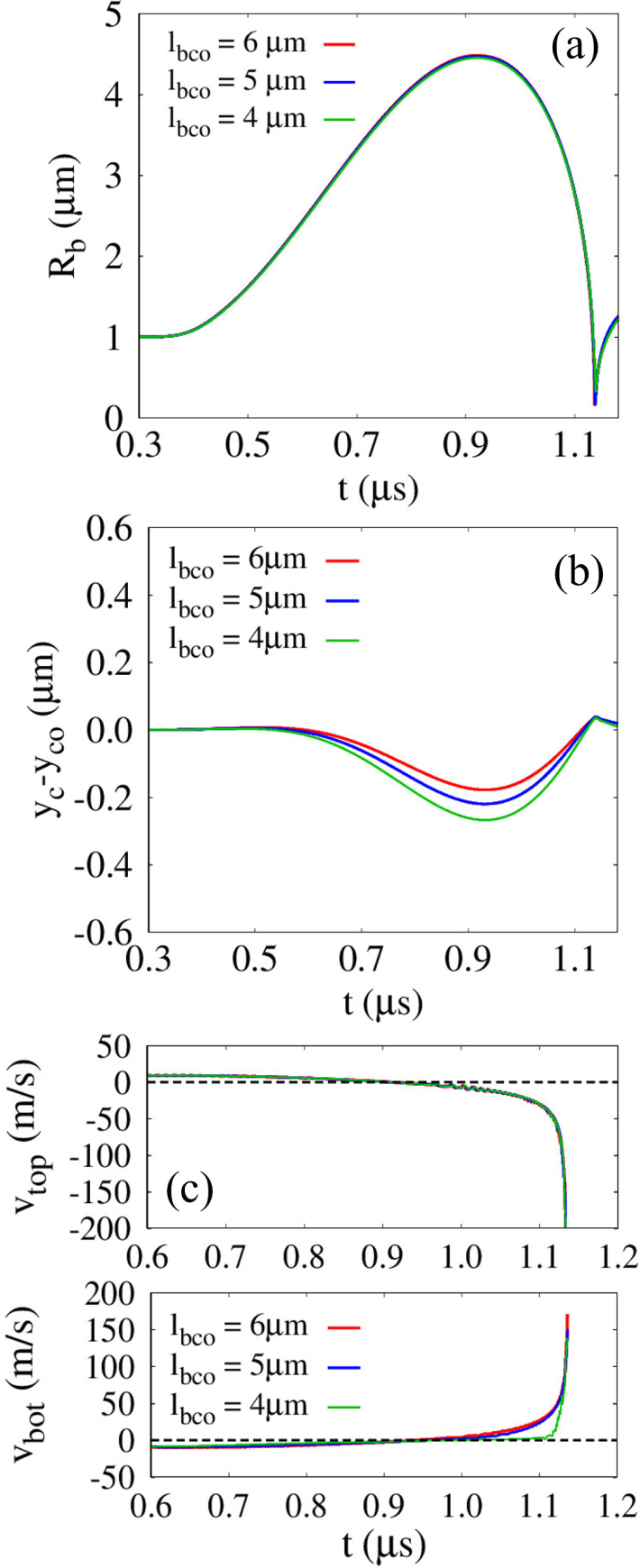
Effect of the distance $l_{bco}$ on the bubble-cell interaction for G=100MPa and $R_{co}=5\;\mu m$: (a) bubble radius $R_{b}$, (b) displacement of the solid cell center $y_{c}-y_{co}$, and (c) liquid velocity $v_{top}$ and $v_{bot}$ near the bubble top and bottom. The time histories are presented up to t=1.18μs, corresponding to the pre-collapse and jet-impact stages.

Fig. 6 plots the influence of the initial distance $l_{bco}$ for G=100MPa and $R_{co}=5\;\mu m$. As $l_{bco}$ decreases from 6μm, during the expansion phase, the temporal variation in the averaged bubble radius $R_{b}$ remains nearly unchanged, as shown in Fig. 6(a); moreover, the first maximum expansion radius $R_{b,max1}$ varies by less than 0.1% across all cases. This indicates that the reaction force exerted by the surrounding cell mainly contributes to cell motion as a rigid body, instead of inhibiting bubble expansion. The movable nature of the cell is reflected in the displacement of the cell center, $y_{c}-y_{co}$, where $y_{co}=-7\;\mu m$, as plotted in Fig. 6(b). As the initial distance decreases from $l_{bco}=6\;\mu m$, $y_{c}-y_{co}$ decreases at a faster rate during bubble expansion, in agreement with earlier experimental findings [40], showing that the downward velocity of a rigid sphere decreases as the ratio between the bubble–sphere distance and $R_{b,max}$ increases. During the contraction stage, the rigid cell returns toward its original position due to the attractive bubble–cell interaction. After bubble collapse, $y_{c}-y_{co}$ decreases again owing to the liquid momentum penetrating the bubble, an effect that becomes pronounced for $l_{bco}=4\;\mu m<R_{b,max}^{o}$, where the liquid jet is strongest. Note that the jet velocity reaches 816m/s for $l_{bco}=4\;\mu m$, which is approximately 2.1 and 3.5 times larger than those for $l_{bco}=5\;\mu m$ and 6μm, respectively. This trend is consistent with previous numerical study [41] reporting that the velocity magnitude of liquid jet increases as the ratio between the bubble–rigid curved surface distance and $R_{b,max}$ decreases. Considering that the jet velocity magnitude is governed by the asymmetry of the surrounding flow field, it can be inferred that this asymmetry becomes increasingly pronounced with decreasing $l_{bco}$, as confirmed in Fig. 6(c). The axial liquid velocities near the upper and lower bubble interfaces, $v_{top}$ and $v_{bot}$, along the central axis (r=0) remain nearly identical during the expansion phase, whereas during contraction $v_{bot}$ progressively decreases as $l_{bco}$ becomes smaller, leading to a pronounced asymmetry, particularly for $l_{bco}=4\;\mu m$. These results highlight that effective bubble–cell interaction requires positioning the bubble at a distance smaller than its maximum radius, satisfying $l_{bco}/R_{b,max}^{o}<1$.

### 3.3 Effect of elastic shear modulus

**Fig. 7:**
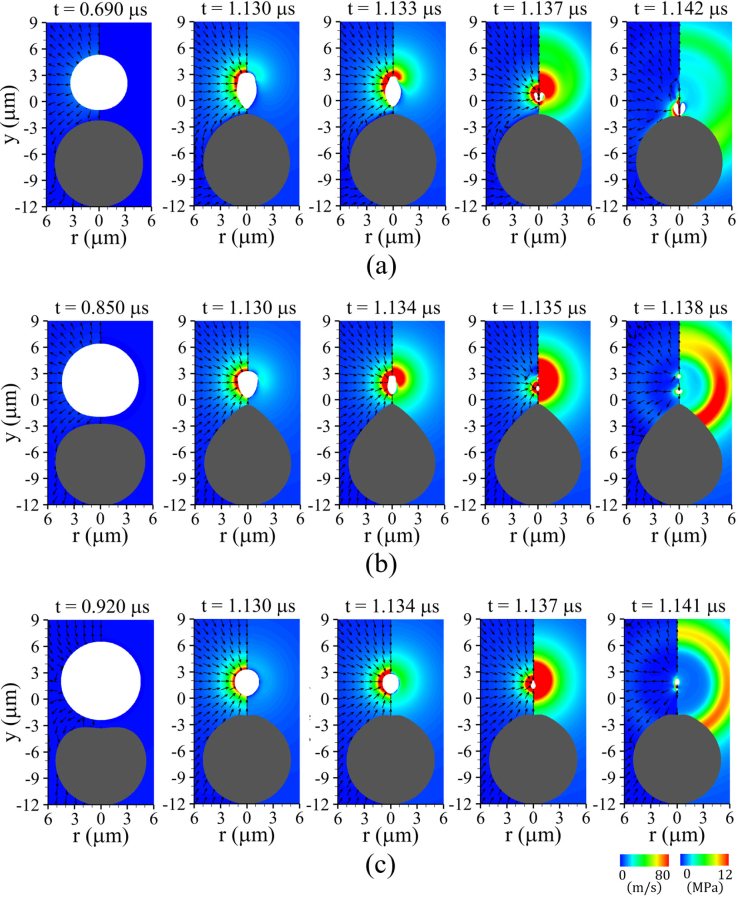
Liquid velocity (left) and pressure (right) fields associated with bubble motion near a deformable cell for $l_{bco}=4\;\mu m$ and $R_{co}=5\;\mu m$, with varying shear modulus: (a) G=1MPa: liquid jet-induced compressive deformation, (b) G=0.1MPa: mushroom-shaped bubble formation and collapse-induced expansive deformation, and (c) G=0.01MPa: expansion-dominated compressive deformation. The white and gray regions indicate the bubble and the solid cell, respectively.

Fig. 7 shows the computed bubble–cell interaction for $l_{bco}=4\;\mu m$ and $R_{co}=5\;\mu m$ as the elastic shear modulus G is progressively reduced from 1 to 0.01MPa. Fig. 7(a) illustrates bubble behavior in the vicinity of an elastic cell with G=1MPa. During the bubble growth phase, the expanding bubble exerts a repulsive hydrodynamic force on the nearby cell through the surrounding liquid, which induces compressive deformation of the cell accompanied by translational motion, as observed at t=0.690μs. In the subsequent collapse phase, attractive bubble–cell interaction produces an inverted cone-shaped bubble, while the cell exhibits a slightly elongated deformation, as illustrated at t=1.130μs. The most pronounced local curvature appears near the top side of the cell and the bottom side of the bubble, which can be attributed to the reduced surface-to-surface distance along the central axis and the resulting strong attractive interaction. Owing to the presence of the elastic cell beneath the bubble, a relatively faster sink flow develops above the bubble, causing preferential collapse of the upper bubble interface, as shown at t=1.133μs. This asymmetric velocity field drives a non-spherical collapse of the bubble, generating a strong liquid jet directed toward the cell, observed at t=1.137μs, followed by direct jet impingement on the cell at t=1.142μs. Unlike the case with a stiffer cell (G=100MPa), the cell with G=1MPa cannot withstand the intense jet impact, resulting in severe localized deformation and the formation of a concave surface, which suggests a potential mechanism for cell perforation. Such severe deformation is consistent with previous numerical study [42] that demonstrated large deformation of polyurea induced by liquid jet impact, along with the associated mesh distortion, using a coupled FEM–BEM approach. These results highlight that cells with moderately large shear modulus are still susceptible to deep local deformation induced by jet impact during ultrasound-driven bubble collapse.

Fig. 7(b) depicts the bubble–cell interaction for a more deformable cell with G=0.1MPa, while all other parameters are kept identical. Compared to the case with G=1MPa, the expanding bubble induces a more pronounced compressive response in the nearby cell, evident at t=0.850μs. For t>0.850μs, the cell begins to deform expansively due to its elastic restoring force and is subsequently pulled rapidly toward the bubble center by the attractive interaction during bubble contraction. This process results in a highly pronounced local curvature near the cell top side along the central axis (r=0), leading to a droplet-like cell shape, which is consistent with the numerical observations of Zevnik and Dular [18], who computed bubble collapse near a bacterial cell while accounting for elastic forces along the bacterial shell. The droplet-like cell geometry generates shear flow parallel to the cell wall, which is directed toward the lower and lateral surfaces of the bubble, as shown at t=1.130μs. The combined effects of the elastic restoring force of the pre-deformed cell and the momentum of the wall-parallel shear flow give rise to a mushroom-like bubble interface shape, as illustrated at t=1.134μs. This behavior is also qualitatively consistent with the numerical observations of Klaseboer and Khoo [43], who reported that perturbations transmitted to the lower bubble interface by elastic repulsion propagate laterally along the bubble surface, resulting in a mushroom-like configuration. Subsequently, the relatively strong radial flow momentum toward the bubble center is converted into the axial direction, as seen at t=1.135μs, leading to axial splitting of the bubble into two parts. After bubble collapse, bidirectional motion of the split bubbles and a stronger shock wave emission than in the G=1MPa case are observed at t=1.138μs, after which the cell gradually recovers its spherical shape due to elastic restoring forces. Such collapse dynamics are consistent with previous experimental observations [44], in which high-speed imaging showed that bubble collapse near a soft membrane leads to neck formation and subsequent two-way splitting into upper and lower bubbles.

Fig. 7(c) shows the bubble-cell interaction for a soft cell with G=0.01MPa. Owing to the weak elastic repulsive force of the soft cell, the cell deforms passively following bubble expansion, with the compressive deformation peaking at t=0.920μs, coincident with the bubble’s maximum volume. During the subsequent bubble collapse phase, the surrounding cell recovers toward its original shape from the deformation induced by the expanding bubble. This weak elastic response leads to the formation of an almost symmetric flow field around the bubble, as observed for 1.130μs≤t≤1.137μs, which promotes a nearly spherical bubble collapse. Compared to the cases with G≥0.1MPa, the reduced asymmetry of the surrounding flow field suppresses bubble collapse toward the cell surface and prevents the formation of a liquid jet, as shown at t=1.141μs. This behavior is consistent with the numerical results in our previous study [15], which demonstrated that bubble collapse near a fluid-like tissue layer with G=0.01MPa primarily results in shock wave emission without the generation of a distinct liquid jet. Although no liquid jet is generated for G=0.01MPa, the pronounced expansive deformation is accompanied by transient tensile loading of the cell membrane, suggesting a potential cell damage mechanism associated with sonoporation-type membrane permeabilization rather than jet-induced perforation. The results in Fig. 7 collectively highlight that ultrasonic bubble motion and the associated cell damage mechanisms are highly sensitive to the elastic properties of spherical cells.

**Fig. 8:**
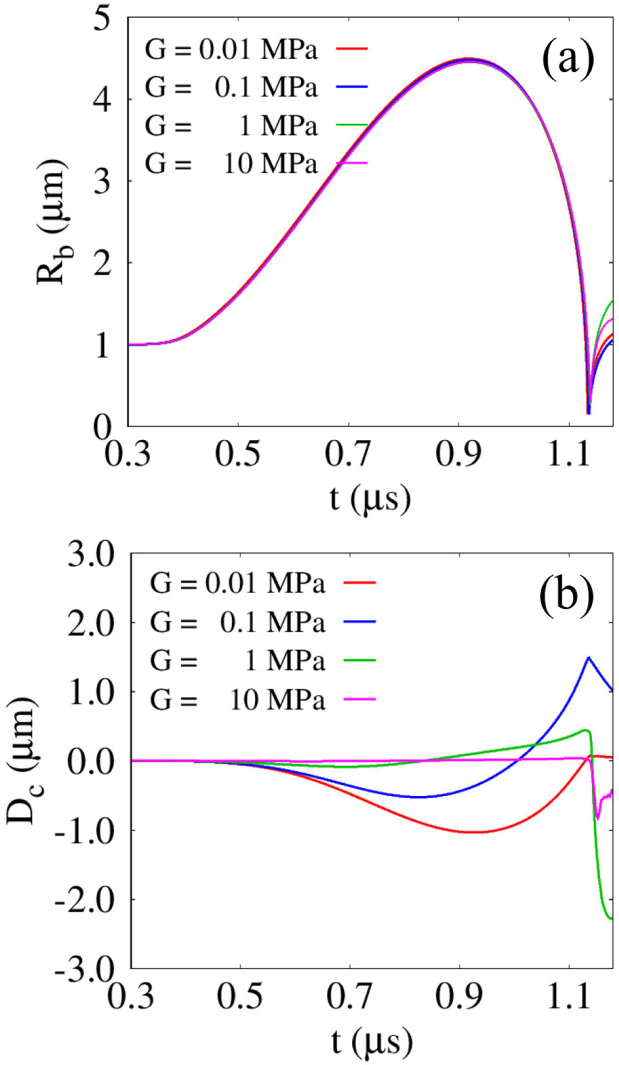
Effect of G on the bubble-cell interaction for $l_{bco}=4\;\mu m$ and $R_{co}=5\;\mu m$: (a) bubble radius $R_{b}$, and (b) cell deformation $D_{c}$. The time histories are presented up to t=1.18μs, corresponding to the pre-collapse and jet-impact stages.

Fig. 8 illustrates the effects of G on bubble and cell dynamics for $l_{bco}=4\;\mu m$ and $R_{co}=5\;\mu m$. As G decreases from 10MPa, the overall variation of $R_{b}$ gradually increases, but the deviation among different cases is still relatively small, as plotted in Fig. 8(a). The corresponding cell deformation pattern induced by bubble dynamics is depicted in Fig. 8(b), where the cell deformation $D_{c}$ is defined as the axial displacement of the cell surface along the central axis (r=0) from the equilibrium spherical position, indicated by the dashed outline in the lower-left inset of Fig. 1, following the definition in previous Ref. [12]. It should be noted that, in the Eulerian framework, solid deformation is resolved using the deformation tensor without tracking the reference configuration of material points, and thus the direct evaluation of strain is not straightforward; in the present axisymmetric simulations, the axial deformation of the cell is dominant, and the axial displacement is therefore adopted as a representative measure of deformation. For G=0.01MPa, $D_{c}$ closely follows the bubble growth phase, exhibiting compressive deformation and attraction, and reaches the first minimum deformation $D_{c,min1}=-1.039\;\mu m$ at t=0.920μs, corresponding to the time of $R_{b,max1}$, before returning toward $D_{c}\approx 0$ during bubble contraction. In contrast, for G=0.1MPa, the cell reaches its first minimum deformation earlier, at t=0.825μs with $D_{c,min1}=-0.527\;\mu m$, and begins to recover during the remaining bubble expansion due to elastic restoring forces. The combined effects of collapse-induced attraction and elastic restoring force subsequently drive the cell to the first maximum deformation $D_{c,max1}$, which is 2.82 times larger than $\left|D_{c,min1}\right|$. This spring-like behavior is consistent with our previous numerical results [45] that calculated the response of a viscoelastic tissue layer with G=0.1MPa to the motion of a closely located acoustic droplet vaporization bubble. Compared to the G=0.1MPa case, a cell with G=1MPa undergoes a pronounced compressive deformation after bubble collapse, reaching a second minimum deformation $D_{c,min2}=-2.378\;\mu m$ at t=1.219μs, which results from the strong liquid jet formed by the substantial repulsive response of a moderately stiff neighboring cell, as observed in Fig. 7(a). Such localized and pronounced cell deformation is qualitatively consistent with the tissue deformation along the central axis (r=0) reported by Moon et al. [20], where the fluid phase was resolved on an Eulerian grid and the viscoelastic solid phase was computed using a Lagrangian framework. For G=10MPa, the jet-induced deformation magnitude $\left|D_{c,min2}\right|$ is reduced by 65.2% compared to the G=1MPa case due to the significantly higher resistance of the cell to deformation, implying that cell deformation can be maximized by liquid-jet impact for moderately large shear moduli. This trend is consistent with the experimental observations [46] reporting that the maximum penetration deformation induced by charge-driven cavitation bubble collapse decreases as the tensile and compressive elastic moduli increase beyond approximately 1MPa. The above results highlight that, depending on the elastic properties of nearby cells, the maximum deformation occurs in either a compressive or expansive manner, accompanied by distinct bubble growth and collapse mechanisms.

### 3.4 Combined effects of cell size, shear modulus and bubble-cell distance

**Fig. 9:**
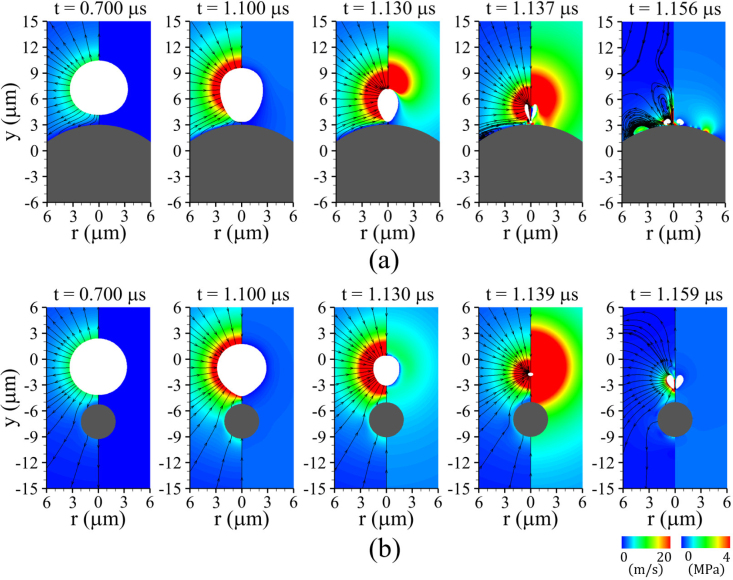
Liquid velocity (left) and pressure (right) fields during bubble collapse near a rigid spherical cell for G=100MPa and $l_{bco}=4\;\mu m$, with varying cell size: (a) $R_{co}=10\;\mu m$: strong liquid jet impact inducing localized deformation, and (b) $R_{co}=2\;\mu m$: insufficient jet development leading to weakened deformation. The white and gray regions indicate the bubble and the solid cell, respectively.

Fig. 9 shows the ultrasonic bubble dynamics located at a distance $l_{bco}=4\;\mu m$ from a rigid solid cell with a large shear modulus of G=100MPa. The numerical results for a large rigid cell with an initial radius of $R_{co}=10\;\mu m$ are presented in Fig. 9(a). During the bubble growth stage, the surrounding liquid source flow exhibits a reduced velocity magnitude near the lower side of the bubble, as observed at t=0.700μs, due to the repulsive effect induced by the rigid cell surface. During bubble contraction, the sink-type liquid flow is also significantly weakened near the lower region of the bubble for 1.100μs≤t≤1.130μs, as the bubble–cell attraction is distributed over a relatively large cell surface area. This results in a pronounced asymmetry between the upper and lower flow fields, which subsequently leads to bubble contraction and liquid jet formation at a location immediately adjacent to the cell surface, as observed at t=1.137μs. This behavior is attributed to the enlarged effective repulsion and attraction area associated with a larger $R_{co}$, consistent with the theoretical interpretation [47], which identified the area of attraction as a key factor governing jet formation during the interaction between a cavitation bubble and a fixed rigid sphere. It should be noted that the effective repulsion and attraction areas, which depend solely on $R_{co}$ and $l_{bco}$, may be influenced by cell shape parameters such as aspect ratio and surface irregularity. Owing to the close proximity of the collapse location, the resulting liquid jet carries a large momentum that acts directly near the cell surface until it is dissipated by viscous effects, as shown at t=1.156μs. In contrast, for a smaller cell with $R_{co}=2\;\mu m$, as shown in Fig. 7(b), the reduced repulsion area allows a substantial portion of the streamlines during bubble expansion to bypass the cell surface without being repelled, as observed at t=0.700μs. This enlarged slip region weakens the interaction between the bubble and the cell surface throughout most of the contraction phase, resulting in a relatively weak flow-field asymmetry for 1.100μs≤t≤1.139μs. Consequently, the induced liquid jet is less pronounced and the downward-displacement of the bubble during collapse is reduced, as observed at t=1.159μs, leading to a weaker jet impact on the cell surface. This is consistent with observations from high-speed imaging experiments [48], which reported that, as the curvature of the nearby solid cell($R_{b,max}^{o}/R_{co}$) increases, the bubble migration distance toward the solid surface after the first collapse decreases, leading to a reduced tendency for liquid jet formation. It should be noted that the present study assumes an initially spherical cell geometry. The extent of the slip region may differ when more realistic cell shapes, such as those characterized by aspect ratio, are considered. This implies that further extension of the present numerical framework is required for more practical applications of ultrasonic systems.

**Fig. 10:**
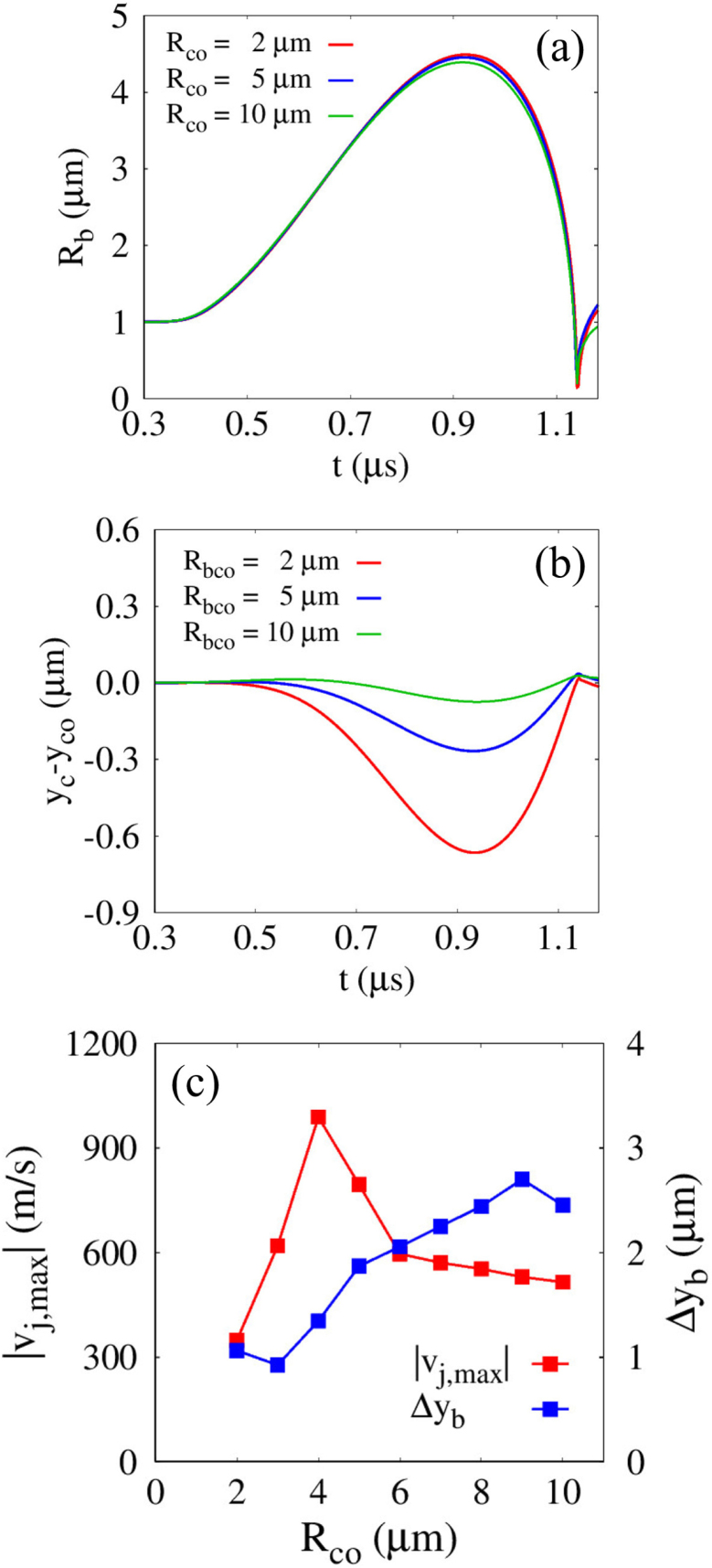
Effect of the cell size $R_{co}$ on the bubble-cell interaction for $l_{bco}=4\;\mu m$ and G=100MPa: (a) bubble radius $R_{b}$, (b) displacement of the solid cell center $y_{c}-y_{co}$, and (c) maximum liquid jet velocity $\left|v_{j,max}\right|$ and the bubble displacement at collapse $\Delta y_{b}$. The time histories are presented up to t=1.18μs, corresponding to the pre-collapse and jet-impact stages.

The influence of $R_{co}$ on $R_{b}$, $y_{c}-y_{co}$, the maximum liquid jet velocity $\left|v_{j,max}\right|$, and the bubble displacement at collapse $\Delta y_{b}$ is presented in Fig. 10 for $l_{bco}=4\;\mu m$ and G=100MPa. As $R_{co}$ increases from 2μm to 10μm, bubble growth is increasingly suppressed by the strong repulsion effect from the cell surface, as shown in Fig. 10(a). With increasing $R_{co}$, the compression force exerted on the nearby cell during bubble expansion also increases; however, the displacement of the cell center $y_{c}-y_{co}$ becomes less sensitive due to the larger cell size and the associated inertia. As a result, the minimum value of $y_{c}-y_{co}$ for $R_{co}=10\;\mu m$ is −0.075μm, which is 88.8% larger than that for $R_{co}=2\;\mu m$, as plotted in Fig. 10(b). The relatively smaller displacement of the larger cell leads to a reduced surface-to-surface distance between the cell and the bubble during the contraction phase. This induces a stronger attractive interaction and a bubble collapse location closer to the cell surface, as observed in Fig. 10(c). As $R_{co}$ increases from 2μm, the bubble displacement at collapse $\Delta y_{b}$ increases gradually and then rises rapidly for $R_{co}\geq 4\;\mu m$. In contrast, the maximum liquid jet velocity $\left|v_{j,max}\right|$ increases rapidly with increasing $R_{co}$ up to $R_{co}=4\;\mu m$, followed by a slight decrease for larger cell sizes. This decreasing trend in jet velocity is consistent with previous numerical observations [41], which reported that the liquid jet velocity reaches its maximum when the radius of a fixed rigid sphere is slightly smaller than $R_{b,max}$ and decreases as the sphere radius further increases. For $R_{co}=10\;\mu m$, $\left|v_{j,max}\right|$ decreases to 515m/s, which is comparable to the value of 430 estimated from the jet velocity correlation reported in numerical study [49] employing Lagrangian simulations for solid deformation. Considering the rapid increase in $\Delta y_{b}$ and the still sufficiently large values of $\left|v_{j,max}\right|$ for $R_{co}\geq 4\;\mu m$, it can be inferred that strong compressive deformation induced by the liquid jet may occur for cells with $R_{co}\approx 4\;\mu m$. This can also be explained by the numerical observations reported in Ref. [49], where Lagrangian simulations showed that the pit depth increases nonlinearly and is highly sensitive to the jet velocity magnitude beyond a certain threshold. This indicates that potential disruption of cells with G≥1MPa can be achieved through jet-induced compressive deformation, whereas cells smaller than a certain critical size may not experience such deformation.

**Fig. 11:**
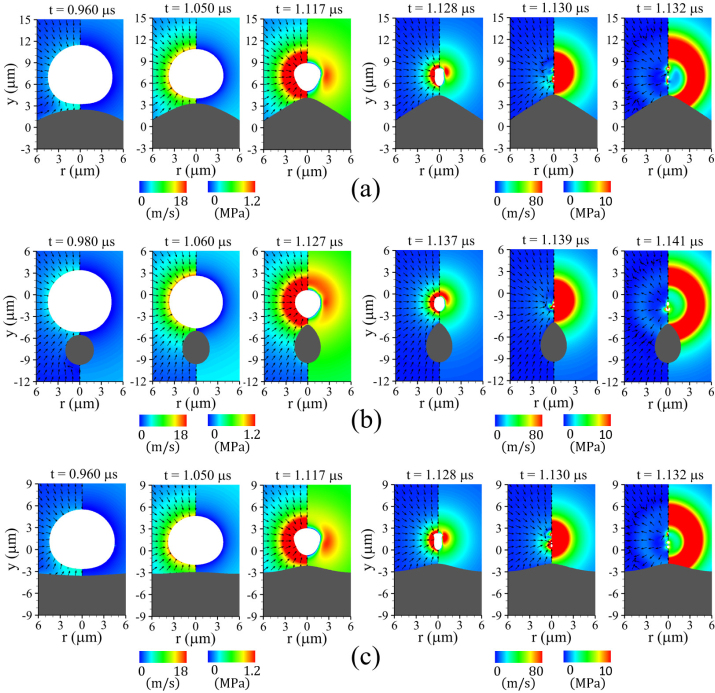
Liquid velocity (left) and pressure (right) fields during bubble collapse near a deformable cell for G=0.1MPa and $l_{bco}=4\;\mu m$, with varying cell size: (a) $R_{co}=10\;\mu m$, (b) $R_{co}=2\;\mu m$, and (c) $R_{co}\to \infty$ (tissue layer), all representing a deformation regime dominated by large-scale expansion. The white and gray regions indicate the bubble and the solid cell, respectively.

Fig. 11 illustrates bubble collapse and the associated liquid flow fields near a deformable solid cell with G=0.1MPa, while varying the cell size $R_{co}$ at a fixed distance of $l_{bco}=4\;\mu m$. During the early stage of bubble contraction, the pre-deformed solid cell with $R_{co}=10\;\mu m$ exerts an elastic restoring force toward the lower bubble surface through momentum transfer, as observed at t=0.960μs in Fig. 11(a). The transferred momentum enhances the contraction velocity at the lower and lateral regions of the bubble, as shown at t=1.050μs, and subsequently induces lateral inflow along the bubble surface at t=1.117μs. During the later stage of contraction, the bubble develops a mushroom-like shape, as reported in Refs. [50], [51], at t=1.128μs, and undergoes axial splitting driven by radial inflow from the sides without forming a liquid jet towards the cell surface. Such a mushroom-like structure is not observed for the smaller deformable cell with $R_{co}=2\;\mu m$, as observed in Fig. 11(b). Compared to the case of large spherical cell with $R_{co}=10\;\mu m$, the momentum transferred to the lower bubble surface from the elastic boundary and the resulting dominant radial inflow are considerably weaker for 0.980μs≤t≤1.127μs. In addition, the enlarged slip area associated with the smaller cell size promotes a strong sink-type flow toward the bubble bottom, inducing the bubble to collapse without generating a liquid jet towards the cell surface. This indicates that the reduced effective repulsion area associated with small cell sizes is insufficient to induce strong deformable cell–bubble interaction. In the limiting case of $R_{co}\to \infty$, corresponding to a planar tissue layer, for which the effective repulsion area is maximized, the overall collapse behavior becomes nearly identical to that observed for $R_{co}=10\;\mu m$, as depicted in Fig. 11(c). These findings suggest that the mushroom-like shape and the associated axial splitting represent distinctive signatures arising from bubble interaction with a deformable solid cell at G=0.1MPa, and further suggest that a strong liquid jet cannot be generated under such low-rigidity conditions regardless of cell size or bubble-cell distance. Instead, compressive deformation of cells with insufficient rigidity but finite elasticity near G=0.1MPa is more likely to be induced by shock-wave effects and bubble migration rather than by liquid jet impact.

**Fig. 12:**
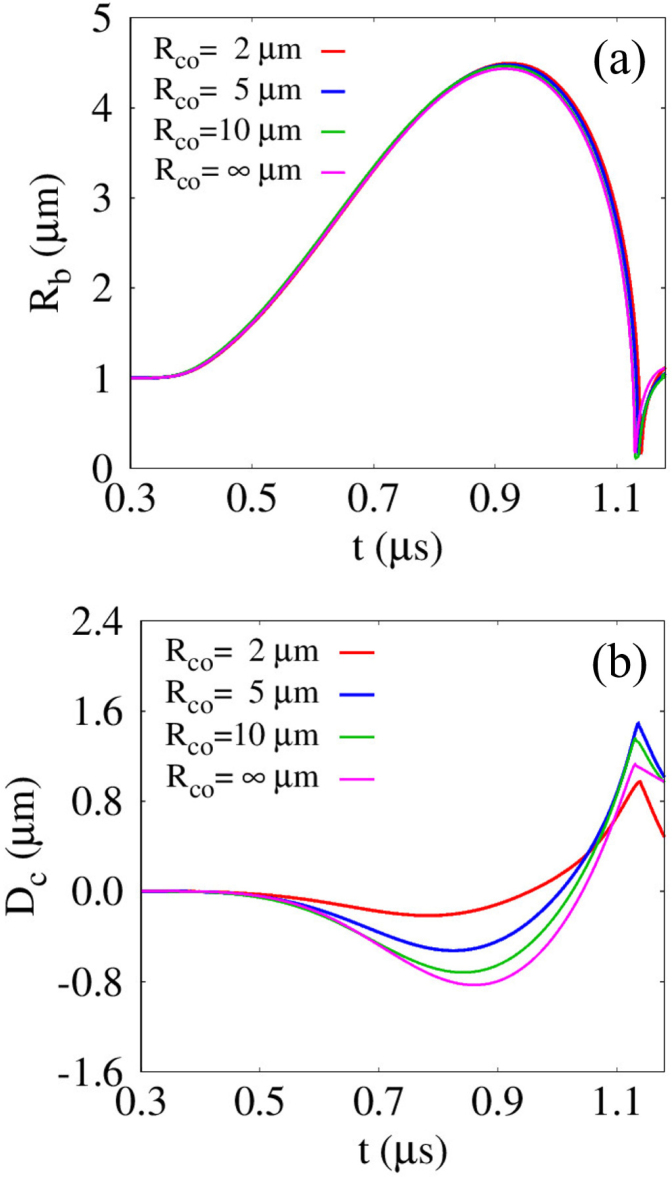
Effect of the cell size $R_{co}$ on the bubble-cell interaction for $l_{bco}=4\;\mu m$ and G=0.1MPa: (a) bubble radius $R_{b}$, and (b) cell deformation $D_{c}$. The time histories are presented up to t=1.18μs, corresponding to the pre-collapse and jet-impact stages.

The influence of $R_{co}$ on $R_{b}$ and $D_{c}$ is presented in Fig. 12 for $l_{bco}=4\;\mu m$ and G=0.1MPa. As $R_{co}$ increases up to 10μm, $R_{b}$ gradually decreases during the first bubble growth–collapse cycle and approaches the limiting trend observed for $R_{co}\to \infty$. In contrast to this reduction in bubble size, the magnitude of the induced cell deformation $\left|D_{c}\right|$ increases with increasing $R_{co}$, as depicted in Fig. 12(b). Accordingly, the magnitude of the first minimum cell deformation $\left|D_{c,min1}\right|$ increases monotonically with $R_{co}$, and the value for $R_{co}=10\;\mu m$ is 2.34 times larger than that for $R_{co}=2\;\mu m$, for which $\left|D_{c,min1}\right|=-0.216\;\mu m$. This trend indicates that increasing $R_{co}$ not only enhances bubble–cell interaction through an enlarged effective repulsion area but also increases cell inertia, making deformational motion more favorable than rigid-body translation. The increase in $\left|D_{c,min1}\right|$ with $R_{co}$ further promotes elastic rebound of the cell, which, when combined with the attractive force during bubble contraction, leads to a maximum expansive deformation $D_{c,max1}$ exceeding $\left|D_{c,min1}\right|$ For $R_{co}=2\;\mu m$, 5μm, and 10μm, the corresponding values of $D_{c,max1}$ are 0.972μm, 1.487μm, and 1.352μm, which are 3.50, 1.82, and 0.88 times larger than $\left|D_{c,min1}\right|$, respectively. Given the absence of liquid jet formation for G=0.1MPa, the cell deformation after reaching $D_{c,max1}$ is gradually relaxed toward its original configuration primarily by elastic restoring forces. A similar spring-like recovery behavior is also observed for $R_{co}\to \infty$, where the lack of liquid jet formation prevents deep and localized compressive deformation. These results suggest that potential perforation or disruption of deformable cells with an elasticity on the order of G=0.1MPa may be achieved through enhancement of the maximum expansive deformation $D_{c,max1}$.

**Fig. 13:**
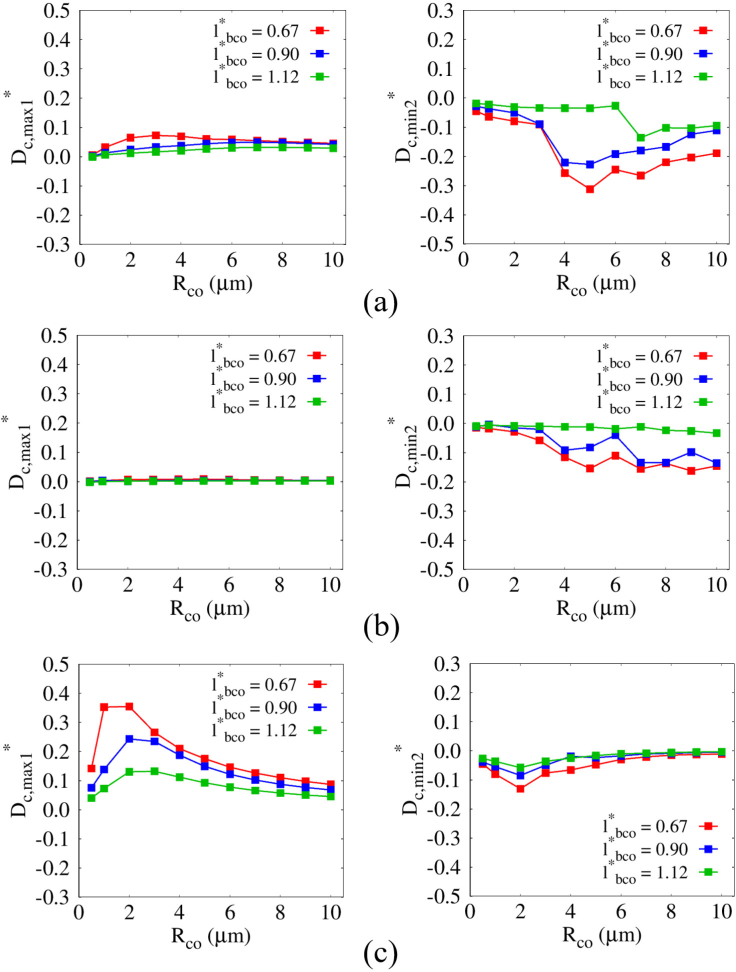
Combined effects of the cell size $R_{co}$ and the normalized distance $l_{bco}^{*}$ on the normalized first maximum cell deformation $D_{c,max1}^{*}$ (left) and the normalized second minimum cell deformation $D_{c,min2}^{*}$ (right) for (a) G=1MPa, (b) G=10MPa, and (c) G=0.1MPa.

Fig. 13 presents the combined effects of cell size and shear modulus on $D_{c,max1}^{*}$ and $D_{c,min2}^{*}$ while varying $l_{bco}^{*}$. Here, $D_{c,max1}^{*}=D_{c,max1}/\left(2R_{co}\right)$ and $D_{c,min2}^{*}=D_{c,min2}/\left(2R_{co}\right)$ are normalized by the cell diameter $2R_{co}$ to quantify the severity of cell deformation. The dimensionless distance is defined as $l_{bco}^{*}=l_{bco}/R_{b,max}^{o}$, where $l_{bco}^{*}=0.67$, 0.90, 1.12 correspond to $l_{bco}=3$, 4, and 5μm, respectively. For G=1MPa, $D_{c,min2}^{*}$ is generally larger in magnitude than $D_{c,max1}^{*}$, as shown in Fig. 13(a). As $R_{co}$ increases, $\left|D_{c,max1}^{*}\right|$ increases accordingly, whereas $\left|D_{c,min2}^{*}\right|$ decreases monotonically and then exhibits an abrupt drop beyond a certain cell size. When the bubble is positioned farther from the cell surface at $l_{bco}^{*}=1.12$, corresponding to $l_{bco}>R_{b,max}^{o}$ $\left|D_{c,min2}^{*}\right|$ shows little variation for $R_{co}<7\;\mu m$ but suddenly reaches $\left|D_{c,min2}^{*}\right|=0.136$ at $R_{co}=7\;\mu m$. This behavior is attributed to the enhanced bubble–cell interaction induced by the enlarged effective reflection area associated with large cell sizes, even when $l_{bco}/R_{b,max}^{o}>1$. For $l_{bco}^{*}=0.90$, $\left|D_{c,min2}^{*}\right|$ remains relatively small for $R_{co}<4\;\mu m$ and then sharply increases to $\left|D_{c,min2}^{*}\right|=0.221$ at $R_{co}=4\;\mu m$, indicating that liquid-jet-induced compressive deformation becomes effective only beyond a critical cell size. A similar trend is observed for $l_{bco}^{*}=0.67$, where $\left|D_{c,min2}^{*}\right|$ remains small for $R_{co}<4\;\mu m$ and reaches $\left|D_{c,min2}^{*}\right|=0.258$ at $R_{co}=4\;\mu m$. This trend is consistent with our previous discussion in Fig. 10(c), where both $\left|v_{j,max}\right|$ and $\Delta y_{b}$ were shown to act effectively only above a certain cell size of $R_{co}=4\;\mu m$ for $l_{bco}=4\;\mu m$. It should be noted that the abrupt increase in $\left|D_{c,min2}^{*}\right|$ beyond a certain cell size exceeds 10% of the cell diameter $2R_{co}$, indicating a regime of significant deformation that may be associated with cell damage. In this study, a threshold parameter $D_{c,cr}^{*}=0.1$ is introduced to identify deformation regimes associated with significant cell deformation. It should be emphasized that $D_{c,cr}^{*}$ does not represent a physical rupture or failure criterion, but rather serves as an indicator for identifying deformation regimes that may be prone to cell damage. This range is consistent with experimental observations [6], [8] in which membrane permeability increases under deformation levels of approximately 10∼30% of the cell diameter $2R_{co}$. These results indicate that large compressive deformation requires the bubble to be positioned closer than a certain bubble-cell distance that depends on the critical cell size, and implies that sufficiently small cells may not experience strong compressive deformation even when the bubble is placed in close proximity. It should be noted that the observed variation in the effective bubble–cell distance with cell size is derived based on single-pulse acoustic conditions. Under the present conditions (G≥1MPa), where liquid-jet formation is the dominant mechanism governing cell deformation, the effective distance for a targeted cell with given size and material properties may be further underestimated compared to that under multi-cycle ultrasonic excitation. For stiffer cells with G=10MPa, the enhanced resistance to deformation leads to overall smaller compressive responses and reduced $\left|D_{c,min2}^{*}\right|$, as shown in Fig. 13(b); in particular, no significant cell deformation is observed over the entire range of $R_{co}$ for $l_{bco}^{*}=1.12$. For $l_{bco}^{*}=0.90$ and 0.67, the cell sizes satisfying $\left|D_{c,min2}^{*}\right|>D_{c,cr}^{*}$ are $R_{co}=7\;\mu m$ and 4μm, respectively, showing an overall increase for G=10MPa compared to G=1MPa This is attributed to the increased resistance to liquid-jet-induced deformation, which requires stronger jet conditions to induce significant cell deformation. Moreover, variations in $l_{bco}$ have a negligible effect on $D_{c,max1}^{*}$, indicating that bubble-induced attraction during contraction is insufficient to induce noticeable deformation for G≥10MPa. In contrast, for highly deformable cells with G=0.1MPa, $D_{c,max1}^{*}$ generally exceeds $\left|D_{c,min2}^{*}\right|$, suggesting that attractive bubble–cell interactions dominate the deformation response, while $\left|D_{c,min2}^{*}\right|$ exhibits minimal variation due to the absence of liquid jet formation, as discussed previously in Fig. 11. As $l_{bco}$ decreases from 5μm to 3μm, $D_{c,max1}^{*}$ increases due to the reduced surface-to-surface distance and the resulting enhancement of bubble–cell interaction. For $l_{bco}^{*}=0.67$, $D_{c,max1}^{*}$ generally exceeds $D_{c,cr}^{*}$, suggesting an increased likelihood of significant localized deformation as the bubble approaches the cell. This trend is qualitatively consistent with deformation trends reported in experimental studies [6], where acoustically excited lipid-stabilized bubbles induce pronounced deformation of histiocytic lymphoma cells when located in close proximity. Overall, these findings highlight that potential disruption of elastic cells with G≥1MPa is primarily governed by deep compressive deformation induced by liquid jets, which requires appropriate tuning of $l_{bco}$ according to both cell size and shear modulus, whereas large deformation of deformable spherical cells with G<1MPa is more likely to be achieved through expansive deformation rather than jet-induced compression.

### 3.5 Effect of ultrasonic pulse amplitude on cell deformation

**Fig. 14:**
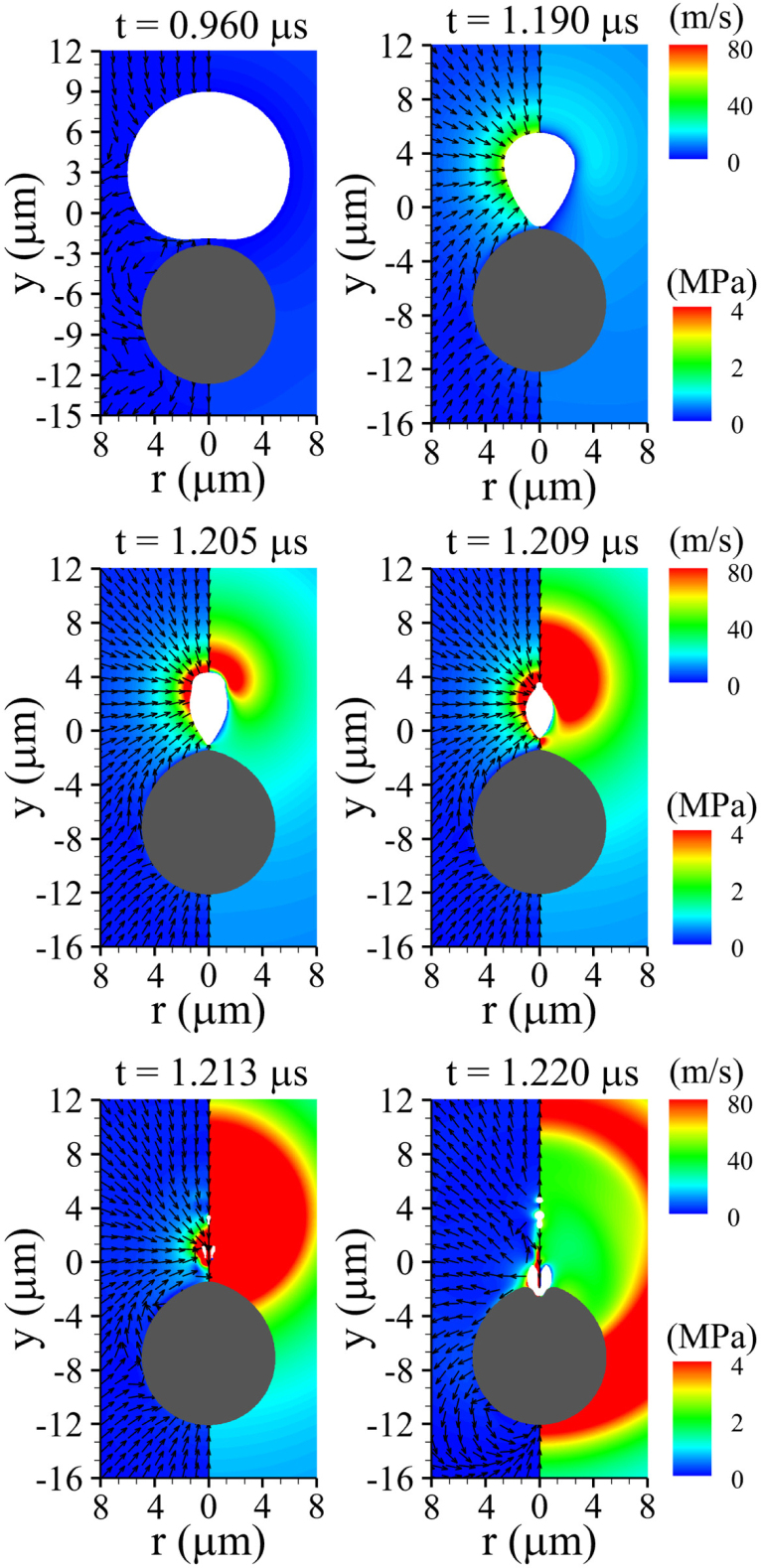
Liquid velocity (left) and pressure (right) fields during bubble collapse near an elastic solid cell under an ultrasonic pulse with $P_{u}=0.4\;\mathrm{MPa}$ for $l_{bco}=5\;\mu m$, G=1MPa and $R_{co}=5\;\mu m$. The white and gray regions indicate the bubble and the solid cell, respectively.

When the pulse amplitude $P_{u}$ increases to 0.4MPa, the numerical results for G=1MPa, $l_{bco}=5\;\mu m<R_{b,max}^{o}$ and $R_{co}=5\;\mu m$ are presented in Fig. 14. Owing to the enhanced pressure difference across the bubble interface under the increased acoustic pressure, the bubble undergoes a larger expansion, which induces the formation of a significantly thin liquid film between the bubble and the cell, as observed at t=0.960μs. The reduced surface-to-surface distance generates strong attractive hydrodynamic interactions between the lower bubble surface and the upper cell surface, leading to substantial retardation of bubble collapse near the cell during the contraction stage. As a result, a pronounced inverted cone-like bubble shape is formed, as shown at t=1.190μs. It is noted that an inverted cone-like bubble shape, which was not observed in the previous results shown in Fig. 5(a) for $l_{bco}=5\;\mu m$, appears in the present computations due to the condition $l_{bco}/R_{b,max1}<1$. During further contraction, the preferential collapse of the upper bubble surface produces a highly asymmetric flow field, as illustrated for 1.205μs≤t≤1.209μs, which subsequently leads to a near-wall collapse accompanied by an intensified liquid jet and shock wave toward the cell surface at t=1.213μs. Consequently, the collapsing bubble directly impacts the cell surface and imparts strong jet momentum, as shown at t=1.220μs, which is also consistent with visualization experiments [46], where the penetration depth induced by a strong microjet was observed using a transparent flexible sample. indicating that the ultrasonic pressure pulse amplitude is a important parameter governing the magnitude of cell deformation through the enhancement of liquid jetting and shock wave intensity.

**Fig. 15:**
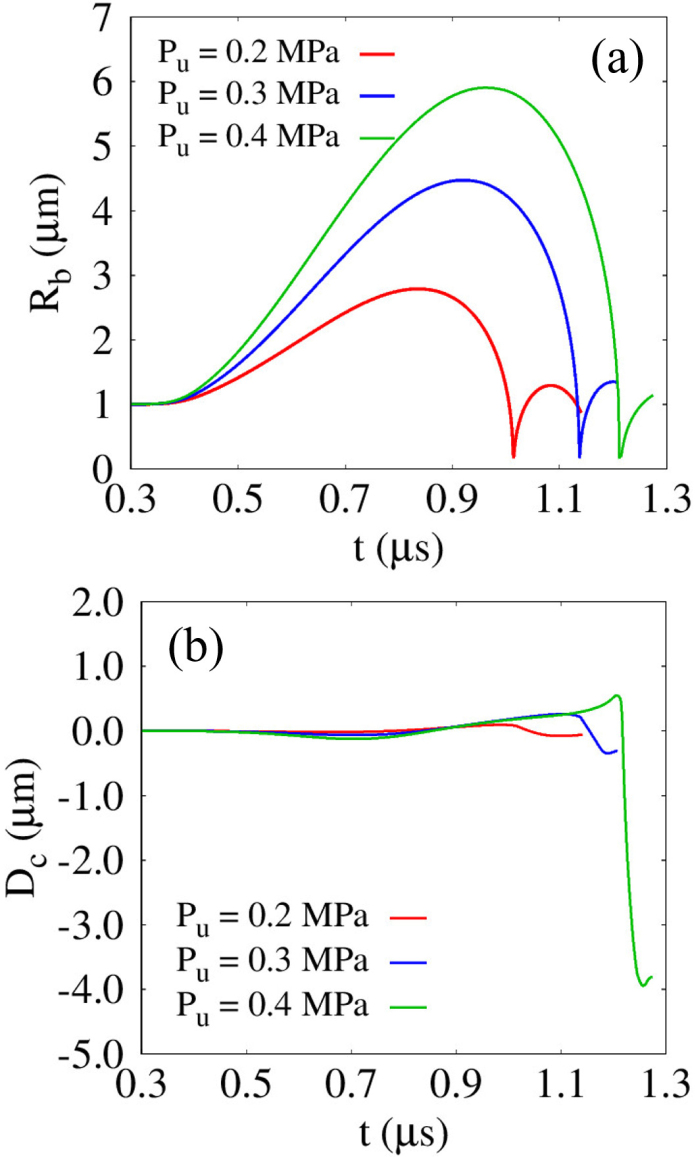
Effect of the pulse amplitude $P_{u}$ on the bubble-cell interaction for $l_{bco}=5\;\mu m$, G=1MPa, and $R_{co}=5\;\mu m$: (a) bubble radius $R_{b}$, and (b) cell deformation $D_{c}$. The time histories are presented up to t=1.14μs, 1.21μs, and 1.28μs for $P_{u}=0.2$, 0.3, and 0.4MPa, respectively, corresponding to the pre-collapse and jet-impact stages.

Fig. 15 plots the effect of $P_{u}$ on $R_{b}$ and $D_{c}$ for G=1MPa, $l_{bco}=5\;\mu m<R_{b,max}^{o}$ and $R_{co}=5\;\mu m$. As $P_{u}$ increases from 0.2MPa to 0.4MPa, the bubble experiences larger expansion and contraction cycles, as plotted in Fig. 15(a). For $P_{u}=0.4\;\mathrm{MPa}$, the expanding bubble reaches a delayed maximum radius of $R_{b,max1}=5.90\;\mu m$ at t=0.962μs, compared to the cases with $P_{u}\leq 0.3\;\mathrm{MPa}$, which is attributed to the enhanced expansion rate and the inertia of the surrounding liquid flow induced during the growth phase. Nevertheless, the corresponding variation in $D_{c}$ remains limited and is primarily governed by the preferred translational motion of the cell rather than localized deformation, as shown in Fig. 15(b). After bubble contraction and collapse, the liquid jet induces a compressive change in $D_{c}$; however, for $P_{u}\leq 0.3\;\mathrm{MPa}$, no pronounced deformation is observed at the cell surface. In contrast, for $P_{u}=0.4\;\mathrm{MPa}$, the minimum deformation reaches $D_{c,min2}=-3.94\;\mu m$, representing the most significant response among the cases considered. This result suggests that, even under conditions where cell disruption does not occur, increasing the ultrasonic pressure amplitude can promote liquid-jet-induced cell disruption.

**Fig. 16:**
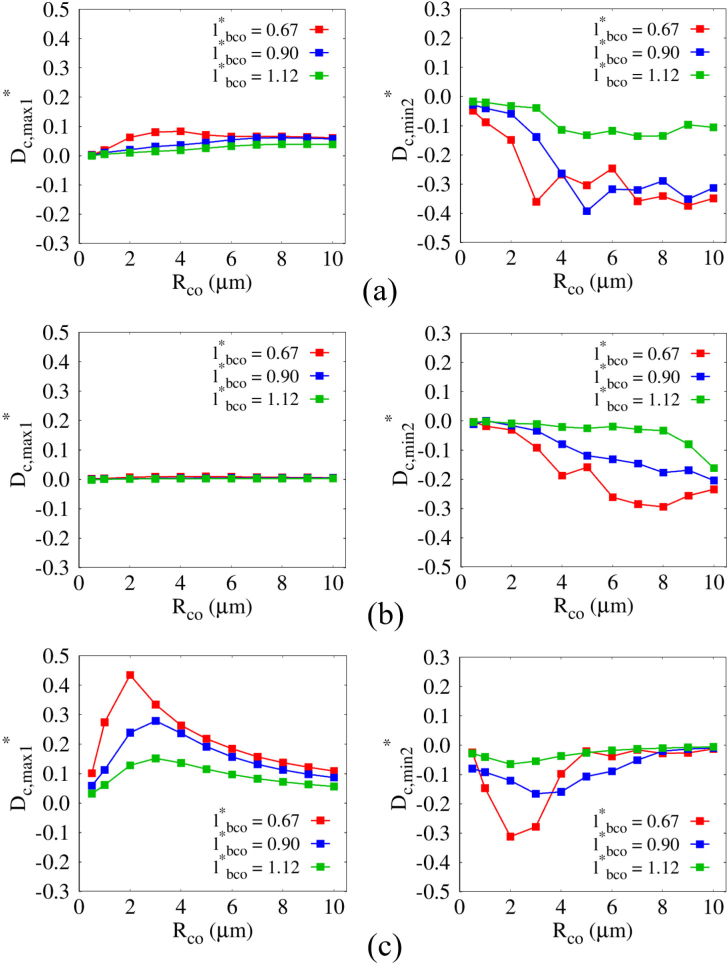
Combined effects of the cell size $R_{co}$ and the normalized distance $l_{bco}^{*}$ on the normalized first maximum cell deformation $D_{c,max1}^{*}$ (left) and the normalized second minimum cell deformation $D_{c,min2}^{*}$ (right) for $P_{u}=0.4\;\mathrm{MPa}$: (a) G=1MPa, (b) G=10MPa, and (c) G=0.1MPa.

The combined effects of $R_{co}$ and G on the normalized parameters $D_{c,max1}^{*}=D_{c,max1}/2R_{co}$ and $D_{c,min2}^{*}=D_{c,min2}^{*}/2R_{co}$ are presented in Fig. 16 by varying the normalized bubble–cell distance $l_{bco}^{*}$ for higher $P_{u}$ of 0.4MPa, where $l_{bco}^{*}=0.67$, 0.90, and 1.12 correspond to $l_{bco}=4.0\;\mu m$, 5.3μm, and 6.6μm, respectively. For G=1MPa, $D_{c,max1}^{*}$ increases with increasing $R_{co}$ and decreasing $l_{bco}$, as plotted in Fig. 16(a), because both a reduced bubble–cell distance and a larger cell size contribute to an increased reflection area. Consistent with the observations in Fig. 13, $\left|D_{c,min2}^{*}\right|$ is generally larger than $D_{c,max1}$ for G=1MPa, indicating that momentum transfer induced by the liquid jet is more effective in deforming cells with moderate elastic properties. For $l_{bco}^{*}=1.12$, the influence of the liquid jet becomes noticeable only for $R_{co}\geq 4\;\mu m$. Nevertheless, the deformation range associated with potential cell damage remains broader than that in Fig. 13, indicating that a higher pressure pulse amplitude and the resulting intensified bubble collapse can still induce deformation levels that may lead to significant deformation even for smaller cell sizes. It should be noted that potential cell damage can be enhanced by increasing the number of acoustic pulse cycles, in addition to increasing $P_{u}$. Even when the initial collapse does not generate a sufficiently strong liquid jet to induce significant deformation, the bubble can migrate toward the cell surface, thereby reducing the bubble–cell distance and enabling stronger interaction during subsequent cycles. Under conditions where jet-induced deformation is dominant (G≥1,MPa), sustained ultrasonic excitation can lead to significant compressive deformation of the cell. In contrast, for highly deformable cells where bubble migration toward the cell surface is suppressed, compressive deformation is unlikely to develop regardless of the number of acoustic cycles. For the closest distance ($l_{bco}^{*}=0.67$), $D_{c,min2}^{*}$ exhibits an abrupt reduction to $R_{co}\geq 3\;\mu m$, indicating a substantially broadened range of cell sizes undergoing effective deformation compared with the size range ($R_{co}\geq 4\;\mu m$) identified in Fig. 13 for $l_{bco}^{*}=0.67∼0.90$. For smaller cells with $R_{co}<3\;\mu m$, however, the deformation remains still limited. This observation suggests that, even for cell sizes where large deformation is not readily induced, an increased ultrasonic pressure amplitude can enhance liquid-jet-induced cell disruption. It is also noted that, particularly for biomedical applications where excessively high pressure amplitudes are generally avoided, controlled adjustment of the bubble–cell distance may provide an effective means for regulating deformation levels potentially associated with cell damage. The variation of $\left|D_{c,min2}^{*}\right|$ becomes more gradual for G=10MPa, as shown in Fig. 14(b), due to the enhanced resistance of stiffer cells to flow-induced deformation. For $R_{co}>8\;\mu m$ the normalized distance $l_{bco}^{*}=1.12$ is insufficient to induce significant cell deformation. It should be noted, however, that for a cell with $R_{co}=8\;\mu m$ large deformation may still occur under multi-cycle ultrasonic excitation. In contrast, for highly deformable cells with G=0.1MPa, $\left|D_{c,min2}\right|$ is generally smaller than that for G≥1MPa, whereas $D_{c,max1}$ is relatively larger, suggesting that compressive deformation becomes the dominant mechanism governing deformation levels associated with potential membrane damage in this regime. Considering that the bubble–cell attractive interaction is enhanced by an increased reflection area, $D_{c,max1}$ generally increases with increasing $R_{co}$ and decreasing $l_{bco}^{*}$, a trend similar to that observed in Fig. 13 for $P_{u}=0.3\;\mathrm{MPa}$. This consistency indicates that, although increasing $P_{u}$ amplifies the overall deformation magnitude, the fundamental mechanisms of bubble collapse and liquid jet formation remain unchanged for a given cell rigidity. The present simulations assume isothermal conditions and therefore do not explicitly account for thermal effects associated with bubble collapse. It should be noted that, in high-intensity ultrasound environments, localized temperature rise and cavitation-induced heating may influence bubble dynamics [52], [53], which can in turn affect cell viability and membrane integrity. However, the primary focus of the present study is the mechanical deformation induced by bubble collapse and liquid-jet formation. Incorporating thermal effects and thermo-mechanical coupling would be a meaningful extension of the present framework in future studies.

## 4 Conclusions

In this study, ultrasound-driven bubble motion and the resulting deformation of a nearby viscoelastic spherical cell were numerically investigated using a CLSVOF-based framework extended to compressible three-phase flows and coupled with a full Eulerian formulation for large solid deformation. The numerical framework demonstrated various bubble–cell interaction phenomena, including non-spherical bubble collapse, liquid-jet formation, the evolution of mushroom- and inverted cone-shaped bubbles, bidirectional axial bubble splitting, and droplet-shaped cell deformation as well as large compressive deformation, by systematically varying the initial bubble–cell distance, elastic shear modulus, cell size, and ultrasonic pulse amplitude. The results showed that effective bubble–cell interaction requires the bubble to be located within a distance smaller than the maximum expansion radius of a free bubble under identical ultrasonic conditions. The dominant cell deformation mechanism depends strongly on the elastic shear modulus G, with liquid-jet-induced compressive deformation for stiff cells (G≥1MPa), droplet-shaped cell deformation associated with collapse-induced attraction for deformable cells (G∼0.1MPa), and cell elongation during bubble expansion for fluid-like cells (G≤0.01MPa). In addition, cell size was identified as a key parameter through its influence on the effective repulsion and attraction surface areas, leading to a shift in the critical size range for liquid-jet-induced deformation as a function of the initial bubble–cell distance. Increasing the ultrasonic pressure amplitude was shown to intensify bubble collapse and liquid jetting, thereby enabling effective deformation for smaller cell sizes, although the applicability of excessively high pressure amplitudes may be limited in certain applications. Overall, the present results provide a mechanistic understanding of ultrasound-driven bubble–cell interactions by explicitly linking bubble dynamics, material properties, and size effects, and highlight the central role of controlling the initial bubble–cell distance, together with cell elasticity and size, in regulating cell deformation and damage mechanisms. However, it should be emphasized that the present analysis is restricted to the first oscillation cycle, owing to the lack of grid convergence in the post-collapse regime. Consequently, multi-cycle bubble dynamics and the cumulative effects of repeated bubble oscillations on cell deformation are not captured in this study. Future work should address these limitations to provide a more comprehensive understanding of bubble–cell interactions under realistic ultrasonic conditions. In addition, the present framework should be extended to configurations involving multiple bubbles and cells, where bubble–bubble interactions such as clustering, coalescence, and mutual jetting may significantly influence liquid jet formation and the resulting cell or tissue deformation. Further experimental validation of the present numerical findings is also required, for example, through high-speed imaging of bubble–cell interactions under controlled cell sizes and mechanical properties.

## CRediT authorship contribution statement

**Jaesung Park:** Writing – original draft, Software, Investigation, Conceptualization. **Gihun Son:** Writing – review & editing, Supervision, Methodology, Conceptualization.

## Declaration of competing interest

The authors declare that they have no known competing financial interests or personal relationships that could have appeared to influence the work reported in this paper.
